# New Pim-1 Kinase Inhibitor From the Co-culture of Two Sponge-Associated Actinomycetes

**DOI:** 10.3389/fchem.2018.00538

**Published:** 2018-11-15

**Authors:** Seham S. El-Hawary, Ahmed M. Sayed, Rabab Mohammed, Mohammad A. Khanfar, Mostafa E. Rateb, Tarek A. Mohammed, Dina Hajjar, Hossam M. Hassan, Tobias A. M. Gulder, Usama Ramadan Abdelmohsen

**Affiliations:** ^1^Pharmacognosy Department, Faculty of Pharmacy, Cairo University, Cairo, Egypt; ^2^Pharmacognosy Department, Faculty of Pharmacy, Beni-Suef University, Beni-Suef, Egypt; ^3^Pharmacognosy Department, Faculty of Pharmacy, Nahda University, Beni-Suef, Egypt; ^4^Faculty of Pharmacy, The University of Jordan, Amman, Jordan; ^5^College of Pharmacy, Alfaisal University, Riyadh, Saudi Arabia; ^6^School of Computing, Engineering and Physical Sciences, University of the West of Scotland, Paisley, United Kingdom; ^7^Marine Biodiscovery Centre, University of Aberdeen, Aberdeen, United Kingdom; ^8^Marine Invertebrates, National Institute of Oceanography and Fisheries, Red Sea Branch, Hurghada, Egypt; ^9^Department of Biochemistry, Faculty of Science, Center for Science and Medical Research, University of Jeddah, Jeddah, Saudi Arabia; ^10^Department of Chemistry and Center for Integrated Protein Science Munich (CIPSM), Biosystems Chemistry, Technical University of Munich, Garching, Germany; ^11^Chair of Technical Biochemistry, Technische Universität Dresden, Dresden, Germany; ^12^Department of Pharmacognosy, Faculty of Pharmacy, Minia University, Minia, Egypt

**Keywords:** *Saccharomonospora* sp., *Dietzia* sp., actinomycetes, Saccharomonosporine A, convolutamydine F, docking, Pim-1 kinase, co-cultivation

## Abstract

*Saccharomonospora* sp. UR22 and *Dietzia* sp. UR66, two actinomycetes derived from the Red Sea sponge *Callyspongia siphonella*, were co-cultured and the induced metabolites were monitored by HPLC-DAD and TLC. Saccharomonosporine A (**1**), a novel brominated oxo-indole alkaloid, convolutamydine F (**2**) along with other three known induced metabolites (**3-5**) were isolated from the EtOAc extract of *Saccharomonospora* sp. UR22 and *Dietzia* sp. UR66 co-culture. Additionally, axenic culture of *Saccharomonospora* sp. UR22 led to isolation of six known microbial metabolites (**6-11**). A kinase inhibition assay results showed that compounds **1** and **3** were potent Pim-1 kinase inhibitors with an IC_50_ value of 0.3 ± 0.02 and 0.95 ± 0.01 μM, respectively. Docking studies revealed the binding mode of compounds **1** and **3** in the ATP pocket of Pim-1 kinase. Testing of compounds **1** and **3** displayed significant antiproliferative activity against the human colon adenocarcinoma HT-29, (IC_50_ 3.6 and 3.7 μM, respectively) and the human promyelocytic leukemia HL-60, (IC_50_ 2.8 and 4.2 μM, respectively). These results suggested that compounds **1** and **3** act as potential Pim-1 kinase inhibitors that mediate the tumor cell growth inhibitory effect. This study highlighted the co-cultivation approach as an effective strategy to increase the chemical diversity of the secondary metabolites hidden in the genomes of the marine actinomycetes.

## Introduction

**Figure 1:**
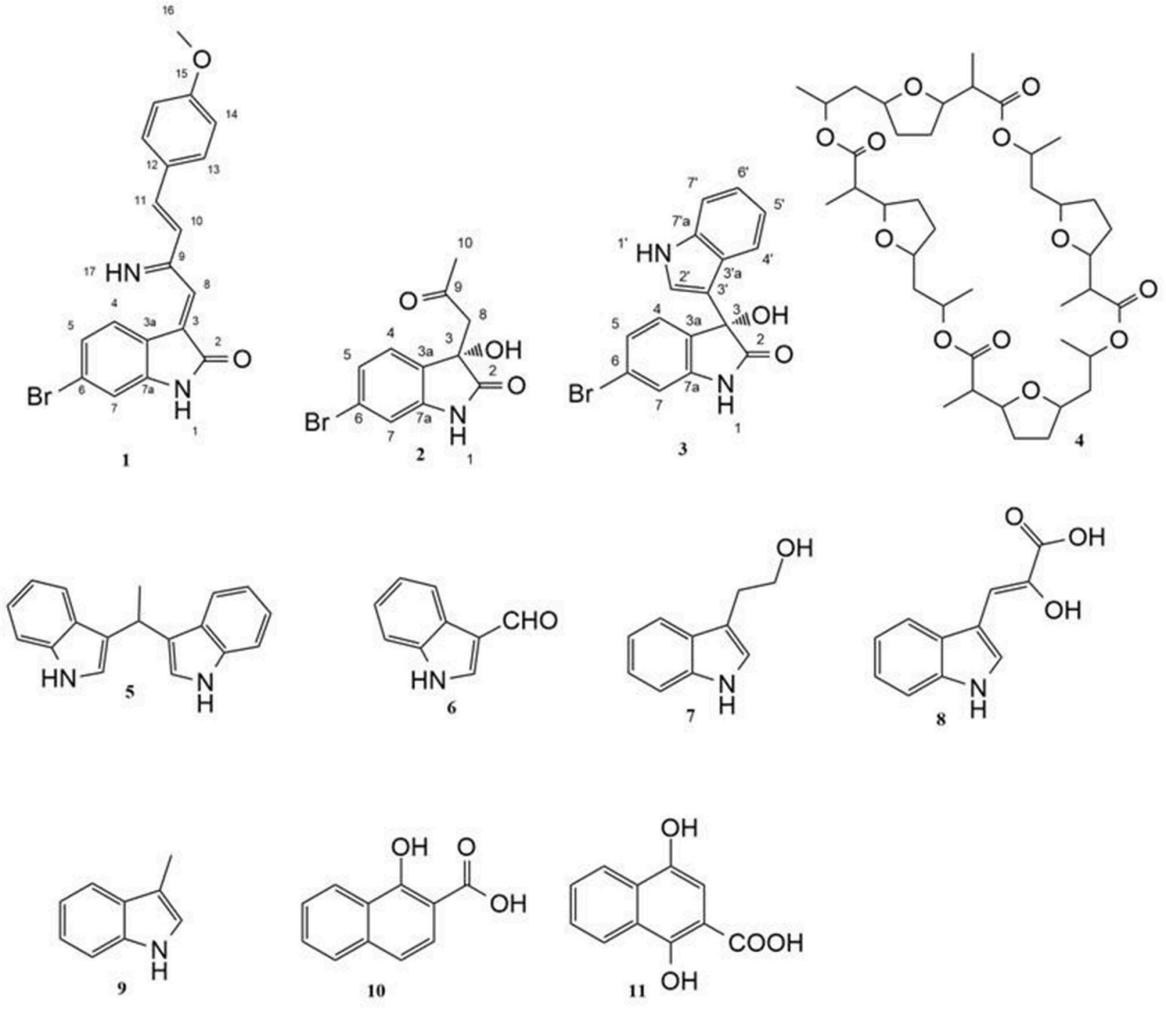
Structures of isolated compounds.

Marine sponge-associated microorganisms have been proved an essential source of biologically active natural products (Thomas et al., 2010; Roue et al., 2012; Abdelmohsen et al., 2014a). Large numbers of secondary metabolites with novel molecular scaffolds and diverse biological activities including antimicrobial (Hentschel et al., 2001; Eltamany et al., 2014), anti-parasitic (Abdelmohsen et al., 2014b; Viegelmann et al., 2014), immunomodulatory (Tabares et al., 2011), and anticancer (Simmons et al., 2011; Yi-Lei et al., 2014) effects have been isolated from sponge-associated actinomycetes. For example, salinosporamide A, a potent inhibitor of the 20S proteasome that has been isolated from a *Salinospora* species (Feling et al., 2003; Gulder and Moore, 2010), entered clinical trials for multiple myeloma treatment, only three years after its discovery (Fenical et al., 2009). Due to the continuous discovery of bioactive natural products from marine microbes, re-isolation of known microbial secondary metabolites has become a real challenge (Hong et al., 2009). However, microbial genome sequencing has confirmed the presence of a large number of silent biosynthetic gene clusters that encode for secondary metabolites which are not produced under normal laboratory conditions (Dashti et al., 2014). Microbial competition for nutrition and other resources is considered one of the most important factors for induction of novel bioactive secondary metabolites (Oh et al., 2005). Crosstalk between microbes inhabiting the same environment induces the unexpressed biosynthetic pathways leading to production of unusual secondary metabolites (Pettit, 2009; Schroeckh et al., 2009; Zuck et al., 2011). Co-cultivation of two different microbial strains together in one culture allows direct interaction between them, which may lead to the induction of new cryptic secondary metabolites not previously detected in the axenic cultures (Rateb et al., 2013). Examples of the production of induced new natural products by co-fermentation of marine derived microorganisms include a rare class of pseurotins, 11-O-methylpseurotin A_2_ derived from mixed fermentation of *Streptomyces bullii* and the fungus *Aspergillus fumigatus* MBC-F1-10 (Rateb et al., 2013), the cyclic depsipeptides emericellamides A and B isolated from a co-culture of marine-derived fungus *Emericella* sp. (CNL-878) and the marine bacterium *Salinispora arenicola* (Oh et al., 2007), the diterpenoids libertellenones A–D isolated from mixed fermentation of the marine α-proteobacterium strain CNJ-328 with the fungus and *Libertella* sp. CNL-52 (Oh et al., 2005) and a chlorinated benzophenone pestalone sourced from a co-culture of the same bacterial strain CNJ-328 with *Pestalotia* sp. strain (Cueto et al., 2001). Recently, co-culture has also proved that both strains affect each other and induce new fungal and bacterial metabolites which were not detected in axenic cultures (Wakefield et al., 2017). In this study, we report on the induction of new bioactive secondary metabolites (Figure 1) saccharomonosporine A (**1**) and convolutamydine F (**2**) along with other three known metabolites **3-5** in response to microbial co-cultivation of two marine actinomycetes, *Saccharomonospora* sp. UR22 and *Dietzia* sp. UR66, derived from the Red Sea sponge *Callyspongia siphonella*. The HPLC and TLC chromatograms of the axenic cultures together with the co-culture derived extracts showed that compounds **1-5** were produced only during co-fermentation of both microbes. On the other hand, fermentation of *Saccharomonospora* sp. UR22 alone led to the isolation of a set of known microbial metabolites (**6-11**). Performing *in vitro* assay and docking studies on the isolated compounds revealed the potential of Pim-1 kinase as a promising target for only compound **1** and **3**. Cytotoxicity evaluation of the isolated compounds showed that compound **1** and **3** have significant antiproliferative activities against HT-29 and HL-60 cell lines. These results coincide with the enzyme inhibition assay ones.

## Material and methods

### General apparatus and chemicals

Ultra violet (UV) spectra were acquired on a ultra-violet visible (UV-vis) spectrometer (Shimadzu UV 1800 spectro, Japan). Optical rotation values were acquired at Bellingham + Stanley ADP600 Series Polarimeter at the sodium D line (589 nm) and 25°C. IR spectra were recorded as KBr disks on a IR spectrophotometer (Shimadzu S8400, Japan) High performance liquid chromatography (HPLC) analysis was performed by Thermofisher dionex ultimate 3000 with PDA detector and Xterra (Waters) C_18_ RP analytical HPLC column (5 μm, 4.6 × 250 mm). High resolution mass spectrometric data were obtained using a Thermo Instruments MS system (LTQ XL/LTQ Orbitrap Discovery) coupled to a Thermo Instruments HPLC system (Accela PDA detector, Accela PDA autosampler, and Accela pump). 1D and 2D NMR spectra were recorded on Bruker Avance III 400 MHz (Bruker AG, Switzerland) with BBFO Smart Probe and Bruker 400 AEON Nitrogen-Free Magnet. Data were analyzed using Topspin 3.1 Software. Each sample was dissolved in suitable deuterated solvent. Chemical shifts were recorded and expressed in ppm related to the TMS signal at 0.00 ppm as internal reference. All solvent used for preparing extracts were of technical grade (ADWIC; El-Nasr Pharmaceutical Chemicals Co., Egypt); and reagents used for preparing samples were of analytical grade (E-Merck, Darmstadt, Germany). Silica gel (60–120 mesh, 50 g, Fluka®) was used for chromatographic isolation and purification. TLC analysis was performed using Merck 9385 pre-coated aluminum plate silica gel (Kieselgel 60) with F254 indicator thin layer plates.

### Isolation and cultivation of the actinomycetes

*Callyspongia siphonella* was collected at a depth of 10 m in the Red Sea (Hurghada, Egypt) in November 2015. A voucher specimen was reserved at the National Institute of Oceanography and Fisheries, Red Sea Branch, Invertebrates Department. Sponge biomass was transferred to plastic bag containing seawater and transported to the laboratory. Sponge specimens were rinsed in sterile seawater, cut into pieces of ca. 1 cm^3^, and then thoroughly homogenized in a sterile mortar with 10 volumes of sterile seawater. The supernatant was diluted in ten-fold series (10^−1^, 10^−2^, 10^−3^) and subsequently plated out on agar plates. Five different media M1 (Mincer et al., 2002), ISP2 medium (Shirling and Gottlieb, 1966), oligotrophic medium (OLIGO) (Olson et al., 2000), actinomycete isolation agar (AIA) (Lechevalier and Lechevalier, 1975) and marine agar (MA) (Weiner et al., 1985) were used for the isolation of actinomycetes. All media were supplemented with 0.2 μm pore size filtered cycloheximide (100 μg/mL), nystatin (25 μg/mL), and nalidixic acid (25 μg/mL) to facilitate the isolation of slow-growing actinomycetes. Cycloheximide and nystatin inhibit fungal growth, while nalidixic acid inhibits many fast-growing Gram-negative bacteria. All media contained DifcoBacto agar (18 g/L) and were prepared in 1 L artificial sea water (NaCl 234.7 g, MgCl_2_.6 H_2_O 106.4 g, Na_2_SO_4_ 39.2 g, CaCl_2_ 11.0 g, NaHCO_3_ 1.92 g, KCl 6.64 g, KBr 0.96 g, H_3_BO_3_ 0.26 g, SrCl_2_ 0,24 g, NaF 0.03 g, and ddH_2_O to 10.0 L) (Lyman and Fleming, 1940). The inoculated plates were incubated at 30°C for 6–8 weeks. Distinct colony morphotypes were picked and re-streaked until visually free of contaminants. *Saccharomonospora* sp. UR22 and *Dietzia* sp. UR66 were cultivated on ISP2 medium. The isolates were maintained on plates for short-term storage and long-term strain collections were set up in medium supplemented with 30% glycerol at −80°C.

### Molecular identification

16S rRNA gene amplification, cloning and sequencing were performed according to Hentschel et al. using the universal primers 27F and 1492R (Lane, 1991). Chimeric sequences were identified by using the Pintail program (Ashelford et al., 2005). The genus-level affiliation of the sequence was validated using the Ribosomal Database Project Classifier. The genus-level identification of all the sequences was done with RDP Classifier (-g 16srrna, -f allrank) and validated with the SILVA Incremental Aligner (SINA) (search and classify option) (Pruesse et al., 2012). An alignment was calculated again using the SINA web aligner (variability profile: bacteria). Gap-only position were removed with trimAL (-noallgaps). For phylogenetic tree construction, the best fitting model was estimated initially with Model Generator. RAxML (-f a -m GTRGAMMA –x 12345 –p 12345 –# 1000) and the estimated model was used with 1,000 bootstrap resamples to generate the maximum-likelihood tree. Visualization was done with Interactive Tree of Life (ITOL).

### Fermentation, extraction, and isolation

Each strain was fermented in 10 Erlenmeyer flasks (2 L), each containing 1 L of ISP 2 (International Streptomyces Project) medium in artificial sea water and incubated at 30°C for 14 days with shaking at 150 rpm. For co-cultivation experiment, 10 mL of 5-day-old culture of *Saccharomonospora* sp. UR22 was inoculated into 15 Erlenmeyer flasks (2 L), each containing 1 L of ISP 2 medium inoculated with 10 mL of 5-day-old culture of *Dietzia*sp. UR66. After fermentation of single cultures and co-culture, filtration was done, and the supernatant was extracted with ethyl acetate (3 × 500 mL) to give the ethyl acetate extracts. These extracts (400 mg for *Saccharomonospora* sp. UR22, 950 mg for co-culture) were fractionated on a Sephadex LH20 (32-64 μm, 100 x 25 mm, Fluka®) column eluting with MeOH/H_2_O (90:10%), to yield six fractions. Fraction Nr. 2 (from co-culture) was subjected to C18 reversed-phase column chromatography (0.04–0.063 mm, 50 × 10 mm, Merck®) with an isocratic elution using acetonitrile/water (40:60) to give five crude compounds **1-5**. Further purification was performed by preparative TLC on a (20 cm × 20 cm) silica gel plates that developed in CH_2_Cl_2_/MeOH/NH_3_ (7:4:0.5, v/v) solvent system. Fraction Nr. 4 (from *Saccharomonospora* sp. UR22) was treated in the same manner to obtain six known compounds **6-11** (Figure 1).

### HPLC analysis

All actinomycete-derived extracts were analyzed by HPLC-DAD. Fifty micro liters (1 mg/mL in acetonitrile) of bacterial crude extracts was injected. Then, isocratic elution was performed with 50% aqueous acetonitrile containing 0.1% trifluoroacetic as amobile phase over 15 min at a flow rate of 1 mL/min and UV detection at different wavelengths (210, 254, 270, and 300 nm).

### Pim-1 kinase assay

The kinase activity was measured following the manufacturer's instructions (HTScan Kinase Assays from Cell Signaling Technology) using staurosporine as a positive control. Different concentrations of test compounds were incubated with 50 ng of Pim-1 kinase enzyme in the reaction buffer [25 mM Tris–HCl pH 7.5, 10 mM MgCl_2_, 0.1 mM Na_3_VO_4_, 5 mM–glycerophosphate, 2 mM dithiothreitol (DTT)] and the ATP/substrate cocktail [200 μM ATP + 1.5 μM biotinylated BAD Ser112 peptide] at room temperature for 30 min. The reaction was stopped by the addition of stop buffer (50 mM EDTA, pH 8) and transferred to a streptavidincoated 96-well plate. After incubation for 1 h at room temperature, the wells were washed three times with phosphate-buffered saline Tween-20 (PBST) buffer. After incubation with anti BADSer112 antibody for 2 h, the wells were washed three times with 1 × PBST and incubated with the peroxidase-conjugated secondary antibody for 30 min. Following three washes with 1 × PBST, the substrate (3,3,5,5-tetramethylbenzidine) was added and the samples were incubated at room temperature for 15 min. The reaction was stopped by the addition of 2 N HCl and the absorbance was measured with a spectrophotometer at 450 nm. The assay was performed in triplicate.

### Docking studies

The crystal structure of Pim-1 of PDB code 3umw was used. Docking experiments were conducted employing LigandFit docking engine. This docking software considers the ligand as flexible and the receptor as rigid structure. The binding site was generated from the “Find sites as volume of selected ligands” option in Discovery Studio 2.5. The number of trials of Monte Carlo search parameters = 30,000; and search step for torsions with polar hydrogens = 30.0°. The root mean square threshold for ligand-to-binding-site shape matching was set to 2.0 Å, employing a maximum of 1.0 binding-site partitions. The interaction energies were assessed employing the CFF force field (v.1.02) with a non-bonded cutoff distance of 10.0 Å and distance-dependent dielectric. An energy grid extending 5.0 Å from the binding site was implemented. The interaction energy was estimated with a trilinear interpolation value using soft potential energy approximations. Rigid body ligand minimization parameters: 40 steepest descent iterations followed by the 80 Broyden–Fletcher–Goldfarb–Shannon minimization iterations were applied to every orientation of the docked ligand. The proposed inhibitors were further energy minimized within the binding site by implementing the “Smart Minimization” option for a maximum of 1,000 iterations (Khanfar and Taha, 2013).

### MTT assay

Cell proliferation was evaluated in cell lines by the MTT assay in triplicates. 10^4^ cells were plated in a 96-well microtiter plate in a final volume of 100 μl of culture medium. Cells were treated for 24 h with test compound at 37 °C with 5% CO_2_. After treatment, the cells were immediately incubated with 10 μl MTT (5.0 mg/mL) for 4 h at 37°C. The cells were then lysed in 100 μl of lysis buffer (isopropanol, conc. HCl and Triton X-100) for 10 min at room temperature and 300 rpm/min shaking. The enzymatic reduction of MTT to formazan crystals that dissolved in DMSO was quantified by photometry at 570 nm. Dose-response curves were generated and the IC_50_ values were define d as the concentration of compound required to inhibit cell proliferation by 50%. 5-Flurouracil was used as a positive control.

## Results and discussion

### Mono and co-culture HPLC profiles

**Figure 2:**
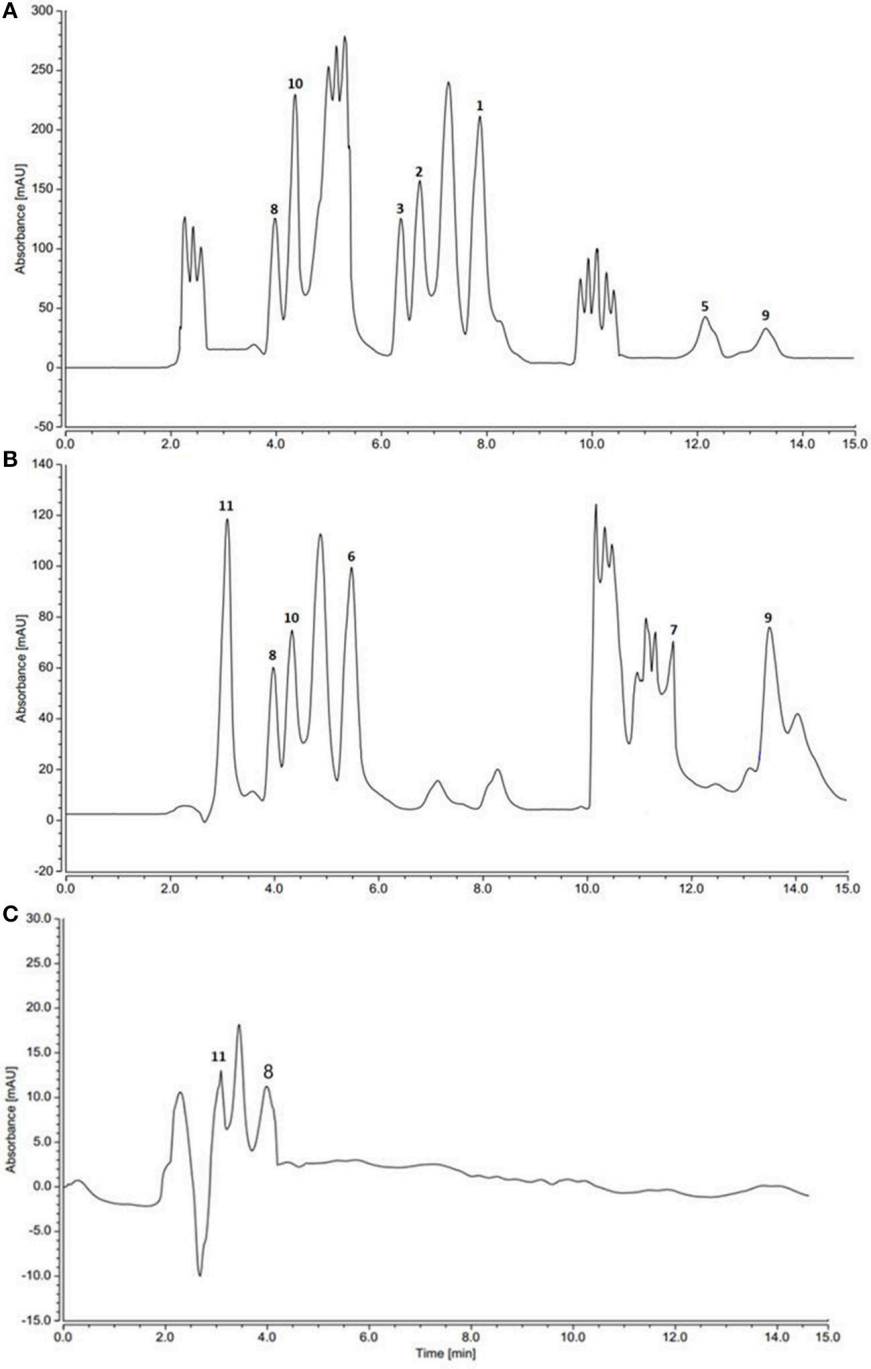
HPLC profiles of actinomycetes extracts. **(A)** *Saccharomonospora* sp. UR22 and *Dietzia* sp. UR66 co-culture **(B)** *Saccharomonospora* sp. UR22 mono-culture **(C)** *Dietzia* sp. UR66 mono-culture.

HPLC and TLC analysis of EtOAc extracts obtained from axenic fermentation of each actinomycete compared to that from mixed fermentation indicated a very different chemical profiles (Figure 2). The co-culture derived extract showed a higher metabolic diversity than extracts from mono cultures. Exclusive detection of metabolites **1-5** in the co-culture illustrated that they are produced only upon co-fermentation of the two microbes.

### Isolation and structural characterization

**Figure 3:**
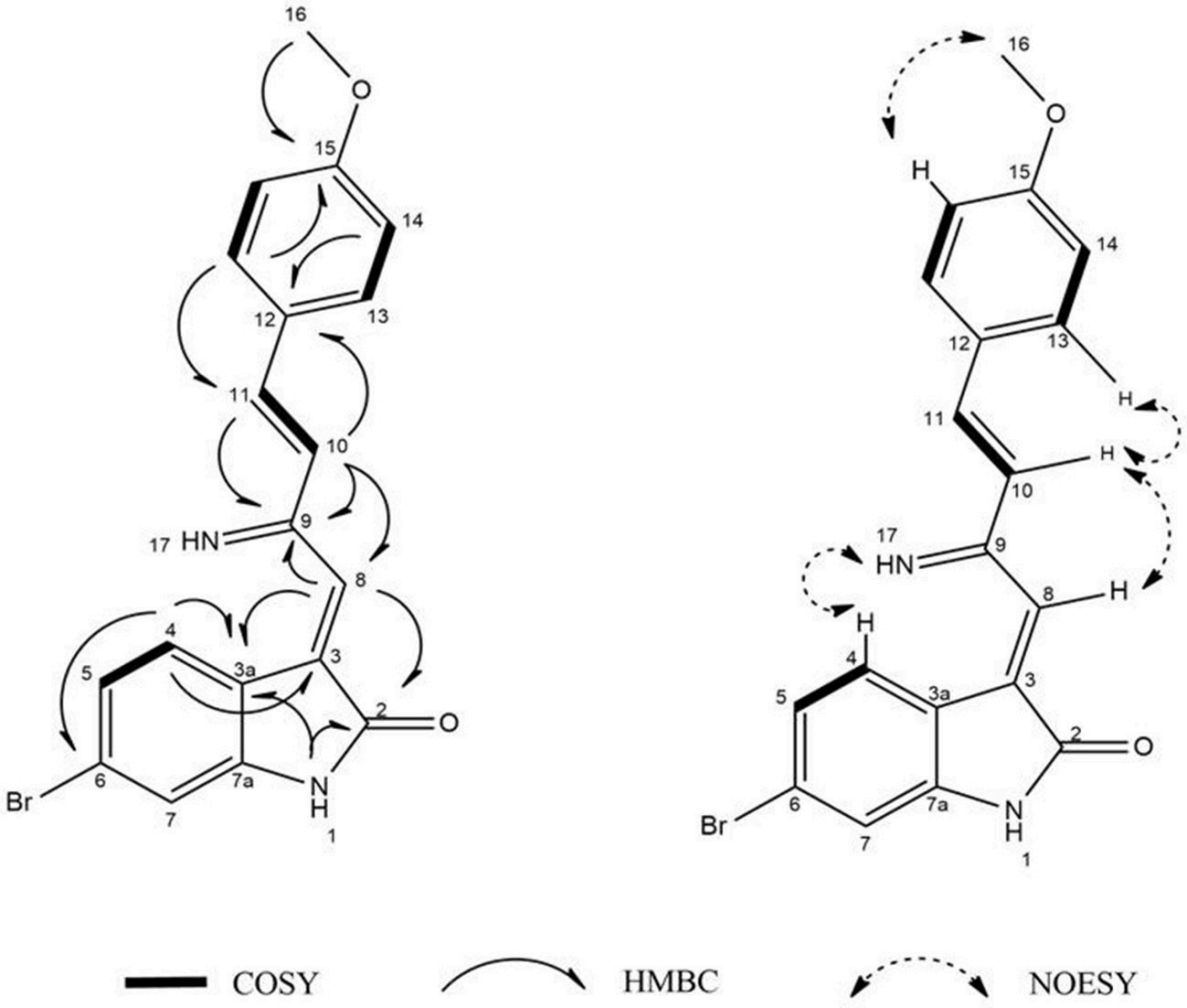
^1^H-^1^H COSY, key HMBC, and NOESY correlations of compound **(1)**.

Guided by HPLC-DAD, the EtOAc extracts from the liquid cultures of the axenic fermentations and mixed fermentation were subjected to Sephadex LH-20 column chromatography followed by C_18_ reversed-phase column chromatography and final preparative TLC purification to target the isolation of the new metabolites **1** and **2** together with the other nine known metabolites **3-11**. Compound **1** was isolated as yellow amorphous powder. The molecular formula C_19_H_15_O_2_N_2_Br was suggested on the basis of positive HRESIMS ion at *m/z* 383.0392 [M+H]^+^, indicating 13 degrees of unsaturation. Furthermore, the presence of a bromine atom was confirmed by its characteristic isotope cluster (Figure S1). ^1^H NMR spectral data of **1** (Figure S2) (Table 1) in DMSO-*d*_6_ suggested the presence of a methyl singlet at δ_H_ 3.81 (3H, s, H-16) and two exchangeable protons (δ_H_ 8.32, 7.98) due to NH. The splitting pattern of resonances at δ_H_ 8.20 (H-7), 8.12 (H-4), and 7.75 (H-5) together with the COSY correlation (Figure S5) between (H-4) and (H-5) suggested the presence of a 1,3,4-trisubstituted benzene ring. On the other hand, the signals at δ_H_ 7.73 (H-13), 7.03 (H-14) and the ^1^H-^1^H COSY correlation between them indicated the presence of 1,4 disubstituted benzene ring. The DEPTQ spectrum (Figure S3) (Table 1) displayed seventeen signals, with eight *sp*^2^ aromatic carbons including eight CH groups and six quaternary carbons, and one *sp*^3^ aliphatic carbon (CH_3_ group). The spectra also revealed an aminocarbonyl at δ_C_168.8 (C-2), an imino carbon at δ_C_ 157.4 (C-9) and one oxygenated carbon at δ_C_ 55.7 (C-16). The assignment of protonated carbons was achieved by the HSQC data (Figure S4). The key HMBC correlations (Figure S6) from H-4 (δ_H_ 8.12) to C-3 (δ_C_ 143), and from the exchangeable proton H-1(δ_H_ 7.98) to the carbonyl carbons C-2 (δ_C_ 168.8) and C-3a (δ_C_ 122.6) together with the previous reported data (Guo et al., 2010) suggested the presence of brominated indolin-2-one skeleton at C-6 (δ_C_ 123.7). The sub-structure of a methoxyphenyl group was deduced from the HMBC correlation between the methyl protons H-16 (δ_H_ 3.81) and C-15 (δ_C_ 160.6). ^1^H-^1^H COSY correlation between H-11 (δ_H_ 7.88) and H-10 (δ_H_ 7.37) together with HMBC correlations from H-11 to C-10 (δ_C_ 126) and C-9 (δ_C_ 157.4), from H-10 to C-9 and C-8 (δ_C_ 118.2) indicated the presence of an imino butenylidene moiety. The *E* configuration of H-11 (δ_H_ 7.88, d) and H-10 (δ_H_ 7.37, d) was deduced from the *J*-values extracted from the ^1^H NMR spectrum (*J* = 16.4 H_z_). The connection of the imino butenylidene moiety to the methoxyphenyl group was established through the HMBC correlation from H-13 (δ_H_ 7.73) to C-11 (δ_C_ 136). Moreover, the HMBC correlations (Figure 3) from H-8 (δ_H_ 7.96) to C-2 (δ_C_ 168.8) and C-3a (δ_C_ 122.6) connected this moiety to the indolin-2-one skeleton at C-3. The chemical shifts of H-8 at δ_H_ 7.96 and H-4 at δ_H_ 8.12 are very characteristic for *E* configuration at the double bond between C-3 and C-9 (Faita et al., 1994). Furthermore, NOESY correlation (Figures S7, S8) between the NH proton H-17 and H-4, and absence of NOESY cross-peak between H-8 and H-4, also pointed to the *E* configuration at the C-3/C-8 double bond. On that basis, the structure of **1** was established as (*E*)-6-bromo-3-((*E*)-2-imino-4-(4-methoxyphenyl)but-3-en-1-ylidene)indolin-2-one, a new secondary metabolite to which were named as **Saccharomonosporine A**.

**Table 1:** ^1^H (400 MHz) and ^13^C NMR (100 MHz) data for saccharomonosporine A (1) in DMSO-*d*_6_.

**Position**	**δ_H_, mult. (*J* in Hz)**	**δ_C_**	**Type**	**COSY**	**HMBC**	**NOESY**
1-NH	7.98, br s				C-3a, C-2
2		168.8	C		
3		143	C		
3a		122.6	C		
4	8.12, d, (8.8)	127.9	CH	H-5	C-6, C-3, C-7a	H-17
5	7.75, dd, (8.8, 2)	130	CH	H-4	C-6
6		123.7	C		
7	8.2, d, (2)	131.1	CH		
7a		149.3	C		
8	7.96, s	118.2	CH		C-3a, C-9, C-2	H-10
9		157.4	C		
10	7.37, d, (16.4)	126	CH	H-11	C-8, C-12, C-9	H-13, H-8
11	7.88, d, (16.4)	136	CH	H-10	C-13, C-9
12		129	C		
13	7.73, d, (8.8)	129.5	CH	H-14	C-11, C-15	H-10
14	7.03, d, (8.8)	114.9	CH	H-13	C-13, C-15, C-12	H-16
15		160.6	C		
16	3.81, s	55.7	CH_3_		C-15	H-14
17-NH	8.3, br s					H-4

**Figure 4:**
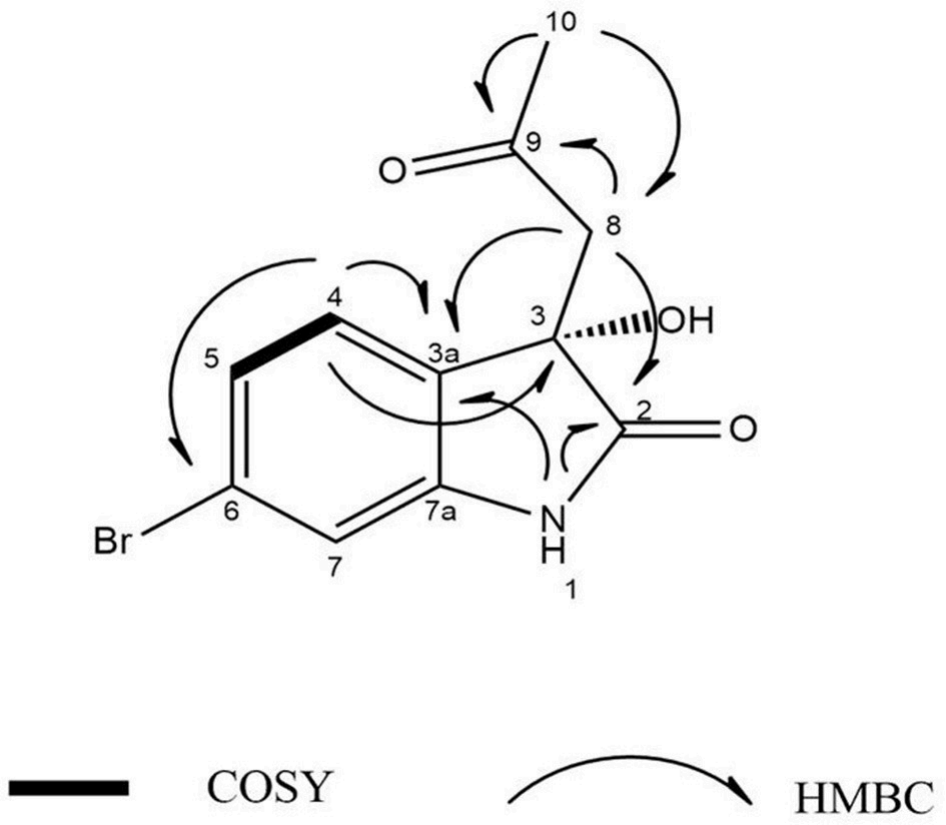
^1^H-^1^H COSY, key HMBC, and NOESY correlations of compound **(2)**.

Compound **2** was isolated as white crystals, the molecular formula C_11_H_10_O_3_NBr was suggested on the basis of positive HRESIMS ion at *m/z* 283.9899 [M+H]^+^, indicating seven degrees of unsaturation. Furthermore, the presence of a bromine atom was confirmed by its characteristic isotope cluster (Figure S9). Similar to compound **1**, the ^1^H and ^13^C NMR spectra (Figures S10, S11) of **2** (Table 2) also revealed an aminocarbonyl at δ_C_178.0 and one oxygenated carbon at δ_C_ 72.3 (C-3). The key HMBC correlations (Figure 4 and Figure S14) from H-4 (δ_H_ 7.19) to C-3 (δ_C_ 72.3), and from the exchangeable proton H-1(δ_H_ 10.37) to the carbonyl carbons C-2 (δ_C_ 178) and C-3a (δ_C_ 131) together with the previous reported data (Guo et al., 2010) suggested the presence of brominated indolin-2-one skeleton. A 2-oxopropyl unit was deduced from the HMBC cross-peaks of H-8 (δ_H_ 3.05) to C-9 (δ_C_ 205.3), H-10 (δ_H_ 2.0) to C-8 (δ_C_ 50), and H-10 to C-9 and was indicated to be attached at C-3 (δ_C_ 72.3) through the HMBC correlation of H-8 to C-3. These spectroscopic data together with the optical rotation value ${\left[\alpha \right]}_{D}^{25}$ –13.8 (*c* 0.85, MeOH), revealed that this compound is the previously unreported (*S*) enantiomer of (*R*)-6-Bromo-3-hydroxy-3-(2 oxopropyl)indolin-2-one, a synthetic 3-hydroxyoxindole derivative (Guo et al., 2010). Several 3-hydroxyoxindole derivatives have been reported from natural marine sources (Kamano et al., 1995; Zhang et al., 1995). For example, convolutamydine A, a brominated (*R*) enantiomer of **2** (Kamano et al., 1995), so the name **convolutamydine F** was suggested to compound **2**.

**Table 2:** ^1^H (400 MHz) and ^13^C NMR (100 MHz) data for 2 in DMSO-*d*_6_.

**Position**	**δ_H_, mult. (*J* in Hz)**	**δ_C_**	**Type**	**COSY**	**HMBC**
1-NH	10.37, br s	-			C-2, C-3a
2	-	178.0	C	
3	-	72.3	C	
3a	-	131.0	C	
4	7.19, d, (9)	125.5	CH	H-5	C-6, C-3, C-3a
5	7.09, dd, (9, 2)	123.8	CH	H-4
6	-	121.6	C	
7	6.93, d, (2)	112.3	CH	
7a	-	144.4	C	
8	3.05, d, (18)	50.0	CH_2_		C-9, C-3a, C-2
	3.33, d, (18)			
9	-	205.3	C	
10	2.0, s	30.4	CH_3_		C-9, C-8
11-OH	6.07, br s	-		

Compound **3** was isolated as white crystals. HRESIMS, 1D and 2D NMR spectral data (Table S1, Figures S15–S20) revealed that compound **3** has a planer structure identical to compound 3f, a previously reported synthetic intermediate (Wang and Ji, 2006), which obtained as a racemic mixture. Compound **3** had a negative optical rotation of ${\left[\alpha \right]}_{D}^{25}$ −8.7 (*c* 0.3, MeOH), in contrast to previous reports on similar optically active compounds that showed a positive optical rotations for the (*R*) enantiomers (Deng et al., 2010; Guo et al., 2010). On this basis compound **3** was identified as (*S*) 6-bromo-3-hydroxy-3-(1H-indol-3-yl) indolin-2-one. To the best of our knowledge, compounds **2** and **3** are considered the first (*S*) 3-hydroxyoxindole derivatives reported from natural sources. Nonactin (**4**) (Wu and Sun, 2006) and vibrindole (**5**) (Ronit et al., 1994) were also isolated from the co-culture of *Saccharomonospora* sp. UR22 and *Dietzia* sp. UR66. In addition, indole-3-carbaldehyde (**6**) (Ashour et al., 2007), tryptophol (**7**) (Gore et al., 2012), indole-3-pyruvic acid (**8**) (Wishart et al., 2007), skatole (**9**) (Chen et al., 2015), 1-hydroxy-2-naphthoic acid (**10**) (Kauko and Lajunen, 1978) and 1,4 dihydroxy-2-naphthoic acid (**11**) (Isawa et al., 2002) were isolated from the axenic culture of *Saccharomonospora* sp. UR22. All isolated known metabolites were identified based on their accurate mass analyses and comparison of their NMR spectroscopic data (Figures S21–S28) with those reported in the literature.

### Pim-1 kinase assay

Pim-1 kinase is a well-established oncoprotein in several tumor entities, e.g., myeloid leukemia, prostate cancer, colorectal cancer, or pancreatic cancer (Weirauch et al., 2013). In addition, over expression of Pim-1 kinase has been described in both human colon adenocarcinoma HT-29 cell (Weirauch et al., 2013) and human promyelocytic leukemia HL-60 (Fan et al., 2016) cell lines. Treatment of the aforementioned cell lines with Pim-1 kinase inhibitors resulted in a potent growth inhibitory activity (Weirauch et al., 2013; Fan et al., 2016). Furthermore, previous compounds structurally similar to compounds **1** and **3** showed potent and selective Pim-1 kinase inhibitory activity (Nakano et al., 2012; Sun et al., 2015). In order to investigate Pim-1 kinase as a potential target that mediates tumor cell growth inhibitory effect, all isolated compounds were evaluated for their efficacy to inhibit the *in vitro* enzymatic activity of Pim-1 kinase (Table 3). Compounds **1** and **3** exhibited significant inhibitory effects on Pim-1 enzyme activity with an IC_50_ value of 0.3 ± 0.02 and 0.95 ± 0.01 μM, respectively.

**Table 3:** *In-vitro* Pim-1 kinase inhibitory activity of isolated metabolites.

**Tested Compound**	**IC_50_ ± S.D. (μM)^a^**
1	0.3 ± 0.02
2	>20
3	0.97 ± 0.01
4	>20
5	>20
6	>20
7	>20
8	>20
9	>20
10	>20
11	>20
Staurosporine	0.04 ± 0.01

a *Values are a mean of 3 independent experiments*.

### Docking of the active compounds

**Figure 5:**
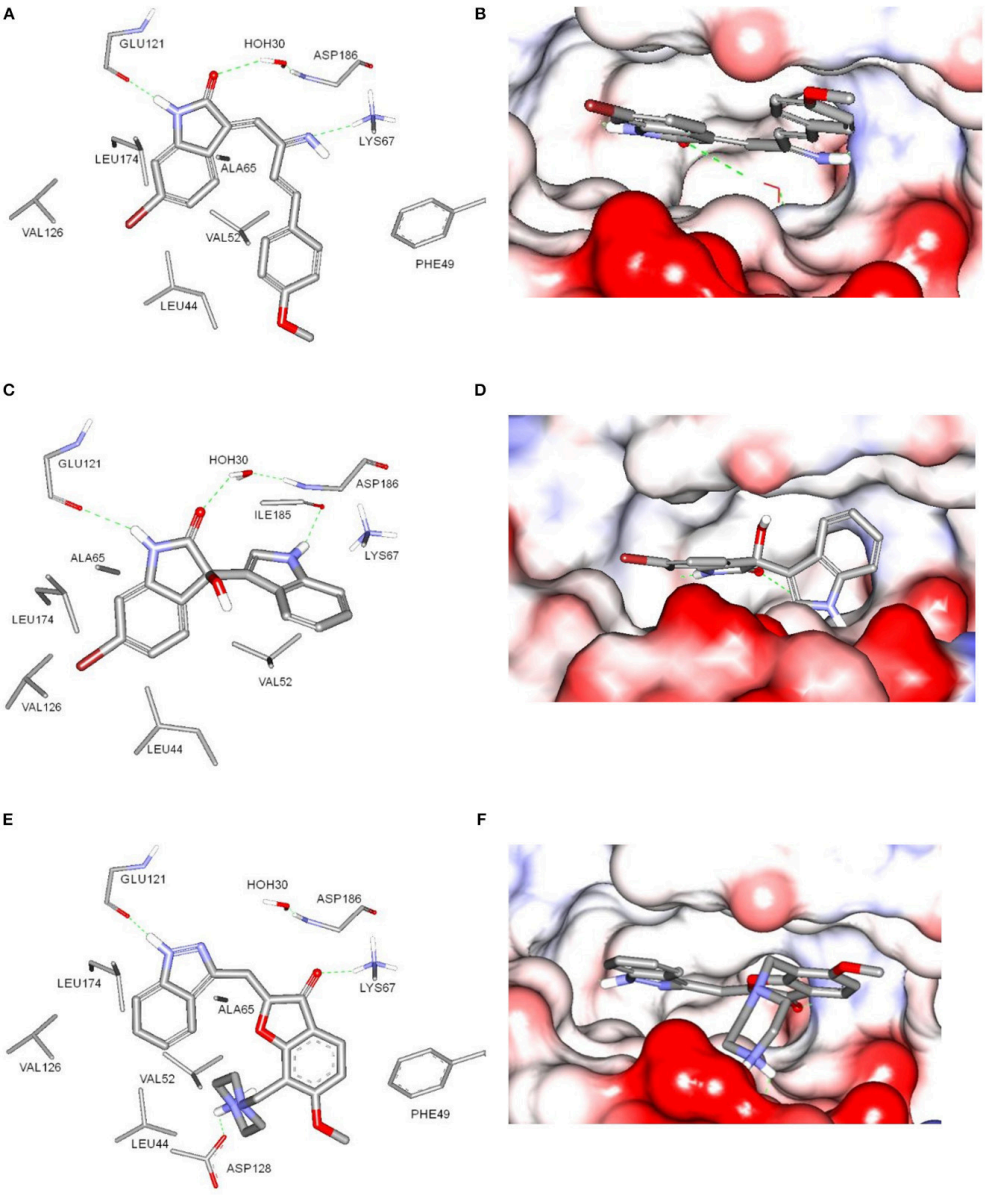
Docking of compounds **1 (A,B)** and **3 (C,D)** within the ATP-binding site of Pim-1 kinase (PDB code 3umw). **(E,F)** The key binding interactions of Pim-1 co-crystallized ligand. The amino acid side chains were depicted in **(A,C,E)** for clarification.

The potential binding mode of compounds **1** and **3** with Pim-1 kinase was analyzed by docking in the ATP-binding site of Pim-1. The protein data bank (PDB) contains several Pim-1 crystallographic proteins, however, Pim-1 of PDB code (3umw) was selected for docking experiments since it has optimum resolution (2.08 Å) and is co-crystalized with Pim-1 inhibitor that is structurally similar to compound **1** and **3** (Nakano et al., 2012). The sphere surrounding the co-crystallized inhibitor was selected as an active site for docking. Both compounds **1** and **3** showed similar docking poses (Figure 5) and comparable to the co-crystalized inhibitor (Nakano et al., 2012). The bromobenzene moiety of compound **1** is impeded within a hydrophobic pocket of ALA65, LEU44, VAL126, and LEU175. The oxindole proton of amidic group is interacted with the hinge region through hydrogen bonding to the main chain carbonyl of GLU121. Hydrogen bonding of this nature was reported with several Pim-1 inhibitors (Nakano et al., 2012). Moreover, the oxindole carbonyl oxygen is hydrogen bonded to the peptidic proton of ASP186 bridged through the conserved water molecule (HOH30). The imine nitrogen of compound **1** is hydrogen bonded to the terminal amino moiety of LYS67. The phenoxy ring is sandwiched between two hydrophobic surfaces of PHE49 and VAL52 (Figure 5A). Alternatively, compound **3** share similar interactions as in compound **1** within the hydrophobic pocket of ALA65, LEU44, VAL126, and LEU175, and hydrogen bonding interaction with GLU121 and ASP186. Moreover, the indole nitrogen is hydrogen bonded with peptidic oxygen of ILE185. However, the interaction with LYS67 is a π-cation interaction with the indole ring of compound **3** (Figure 5C). The binding interactions of compounds **1** and **3** showed high resemblance with the binding mode of the co-crystalized ligand (PDB code: 3umw) within the ATP-binding site of Pim-1 (Nakano et al., 2012). The co-crystalized structure forms similar hydrogen bonding interactions (with GLU121 and LYS67), hydrophobic interactions (with LEU174, VAL126, LEU44, VAL52, and ALA65), and π-stacking (with PHE49) as with compounds **1** and **3** (Figure 5E). However, the co-crystalized ligand forms additional electrostatically-enforced hydrogen bonding interaction with ASP128. Such interaction can explain the superior activity of the co-crystalized ligand (IC_50_ = 3 nM) over compounds **1** and **3**. Therefore, the electrostatic interaction with ASP128 should be taking into consideration for future designing of more active analogs of compounds **1** and **3**.

### Antiproliferative activity

The antiproliferative properties of the nine isolated compounds were evaluated against lung adenocarcinoma H1650, the human promyelocytic leukemia HL-60 and the human colon adenocarcinoma HT-29 cell lines (Table 4). Compounds **1** and **3** displayed potent antiproliferative activity against HL-60 and HT-29 cells (IC_50_ 2.8, 3.6 and 4.2, 3.7 μM, respectively). The other compounds did not exhibit any cytotoxicity at the tested concentrations. These results came in great accordance with the Pim-1 kinase assay results.

**Table 4:** Antiproliferative activity of isolated metabolites against H1650, HL-60, and HT-29 cancer cells.

**Tested Compounds**	**IC_50_ ± S.D. (μM)^a^**		
	**H1650**	**HL-6**	**HT-29**
1	>100	2.8 ± 0.74	3.6 ± 0.55
2	>100	>100	>100
3	>100	4.2 ± 0.23	3.7 ± 0.31
4	>100	>100	>100
5	>100	>100	>100
6	>100	>100	>100
7	>100	>100	>100
8	>100	>100	>100
9	>100	>100	>100
10	>100	>100	>100
11	>100	>100	>100
5-Flurouracil	0.5 ± 0.67	0.2 ± 0.43	0.3 ± 0.35

a *Values are a mean of 3 independent experiments*.

### Data for saccharomonosporine A (1)

Yellow powder (18 mg); UV (MeOH) λ_max_ (log ε) 343 (4.22), 425 (3.16); IR (KBr) ν_max_ 3165, 1716, 1605, 1570, cm^−1^; ^1^H-NMR (DMSO-*d*_6_, 400 MHz); and ^13^C-NMR (DMSO-*d*_6_, 100 MHz) data, see Table 1; HRESIMS *m/z* 383.0392 [M + H]^+^ (calcd for C_19_H_15_O_2_N_2_Br).

### Data for convolutamydine F (2)

White crystals (10 mg); UV (MeOH) λ_max_ (log ε) 323 (3.18), 408 (2.83); ${\left[\alpha \right]}_{D}^{25}$ –13.8 (*c* 0.85, MeOH), IR (KBr) ν_max_ 3331, 1742, 1714 cm^−1^; ^1^H-NMR (DMSO-*d*_6_, 400 MHz) and ^13^C-NMR (DMSO-*d*_6_, 100 MHz) data, see Table 2; HRESIMS *m/z* 283.9899 [M + H]^+^ (calcd for C_11_H_10_O_3_NBr).

## Conclusion

In this study, two sponge-derived actinomycetes, *Saccharomonospora* sp. UR22 and *Dietzia* sp. UR66, were co-fermented in liquid media. The presence of induced metabolites was studied by comparison of the HPLC-DAD and TLC chromatograms of the crude extracts of the two axenic cultures and the co-culture. Co-cultivation of *Saccharomonospora* sp. UR22 and *Dietzia* sp. UR66 induced the biosynthesis of novel oxindole alkaloid saccharomonosporine A (**1**), convolutamydine F (**2**) along with other three induced metabolites (**3-5**) which were not detected in either microorganism in a single culture. Axenic culture of *Saccharomonospora* sp. UR22 led to isolation of common known microbial metabolites **6-11**. Compounds **1** and **3** exhibited potent antiproliferative activities toward HL-60 and HT-29. Based on previous reports on similar compounds, Pim-1 inhibitory assay results and docking studies in the ATP-binding site of Pim-1 kinase, we suggested that both compounds **1** and **3** mediated their cytotoxicity by inhibiting the well-known oncoprotein Pim-1 kinase. These findings highlighted the co-cultivation approach as an effective strategy to enhance the chemical diversity of the secondary metabolites hidden in the genomes of the marine actinomycetes.

## Author contributions

SE, RM, and HH designed the experiments. TM collected and identified the marine sponge. AS, HH, and UA performed the experiments and isolated the compounds. AS and MR performed data acquisition and structure elucidation. DH and TG performed the biological assays. MK performed the docking study. UA, RM, MR, TG, AS and MK drafted and revised the manuscript.

### Conflict of interest statement

The authors declare that the research was conducted in the absence of any commercial or financial relationships that could be construed as a potential conflict of interest.
