# Supplementary material for: New Pim-1 Kinase Inhibitor From the Co-culture of Two Sponge-Associated Actinomycetes
Source: Front Chem. 2018 Nov 15;6:538. doi: 10.3389/fchem.2018.00538 (PMC6262321; doi:10.3389/fchem.2018.00538)
Supplement: Supplementary file 1 [file Presentation_1.PDF]

Supporting Information

New Pim-1 kinase inhibitor from the co-culture of two sponge-associated actinomycetes

Seham S. El-Hawary[1], Ahmed M. Sayed[2,3], Rabab Mohammed[2], Mohammad A. Khanfar,[4,5] Mostafa E. Rateb[2,6,7], Tarek A. Mohammed[8], Dina Hajjar[9], Hossam M. Hassan[2], Tobias A. M. Gulder[10*], and Usama Ramadan Abdelmohsen[11*]

Contents

The ^{1}H (400 MHz) and ^{13}C NMR (100 MHz) data for 3 in DMSO-d_6. 2
The HRESIMS spectrum of compound **1** 3
The ^{1}H NMR (400 MHz, DMSO-d_6) spectrum of **1** 4
The DEPTQ (100 MHz, DMSO-d_6) spectrum of **1** 5
The HSQC (400 MHz, DMSO-d_6) spectrum of **1** 6
The ^{1}H-^{1}H COSY (400 MHz, DMSO-d_6) spectrum of **1** 7
The HMBC (400 MHz, DMSO-d_6) spectrum of **1** 8
The NOESY (400 MHz, DMSO-d_6) spectrum of **1** 9
The NOESY expansion (400 MHz, DMSO-d_6) spectrum of **1** 10
The HRESIMS spectrum of compound **2** 11
The ^{1}H NMR (400 MHz, DMSO-d_6) spectrum of **2** 12
The ^{13}C NMR (400 MHz, DMSO-d_6) spectrum of **2** 13
The HSQC (400 MHz, DMSO-d_6) spectrum of **2** 14
The ^{1}H-^{1}H COSY (400 MHz, DMSO-d_6) spectrum of **2** 15
The HMBC (400 MHz, DMSO-d_6) spectrum of **2** 16
The HRESIMS spectrum of compound **3** 17
The ^{1}H NMR (400 MHz, DMSO-d_6) spectrum of **3** 18
The DEPTQ NMR (400 MHz, DMSO-d_6) spectrum of **3** 19
The HSQC (400 MHz, DMSO-d_6) spectrum of **3** 20
The ^{1}H-^{1}H COSY (400 MHz, DMSO-d_6) spectrum of **3** 21
The HMBC (400 MHz, DMSO-d_6) spectrum of **3** 22
The ^{1}H NMR (400 MHz, $CDCl_3$) spectrum of **4** 23
The ^{1}H NMR (400 MHz, $CDCl_3$) spectrum of **5** 24
The ^{1}H NMR (400 MHz, DMSO-d_6) spectrum of **6** 25
The ^{1}H NMR (400 MHz, $CDCl_3$) spectrum of **7** 26
The ^{1}H NMR (400 MHz, CD_3OD) spectrum of **8** 27
The ^{1}H NMR (400 MHz, $CDCl_3$) spectrum of **9** 28
The ^{1}H NMR (400 MHz, CD_3OD) spectrum of **10** 29
The ^{1}H NMR (400 MHz, CD_3OD) spectrum of **11** 30

Table S1. ^{1}H (400 MHz) and ^{13}C NMR (100 MHz) data for 3 in DMSO-d_6.

Position	$\delta_{H,}$ mult. (J in Hz)	δ_C	Type
1-NH	-	-	
2	-	178.7	C
3	-	178.7	C
3a	-	125.24	C
4	7.17, d, (9)	127	CH
5	7.15, dd, (9, 2)	124.05	CH
6	-	121.98	C
7	7.06, d, (2)	112.94	CH
7a	-	143.92	C
1'-NH	11.05, s	-	
2'	7.07, s	124.79	CH
3'	-	115.16	C
3'a	-	133.3	C
4'	7.4, d, (9)	120.7	CH
5'	6.9, t, (8.8)	119.07	CH
6'	7.05, t, (8.8)	121.62	CH
7'	7.35, d, (9)	112.03	CH
7'a	-	137.27	C

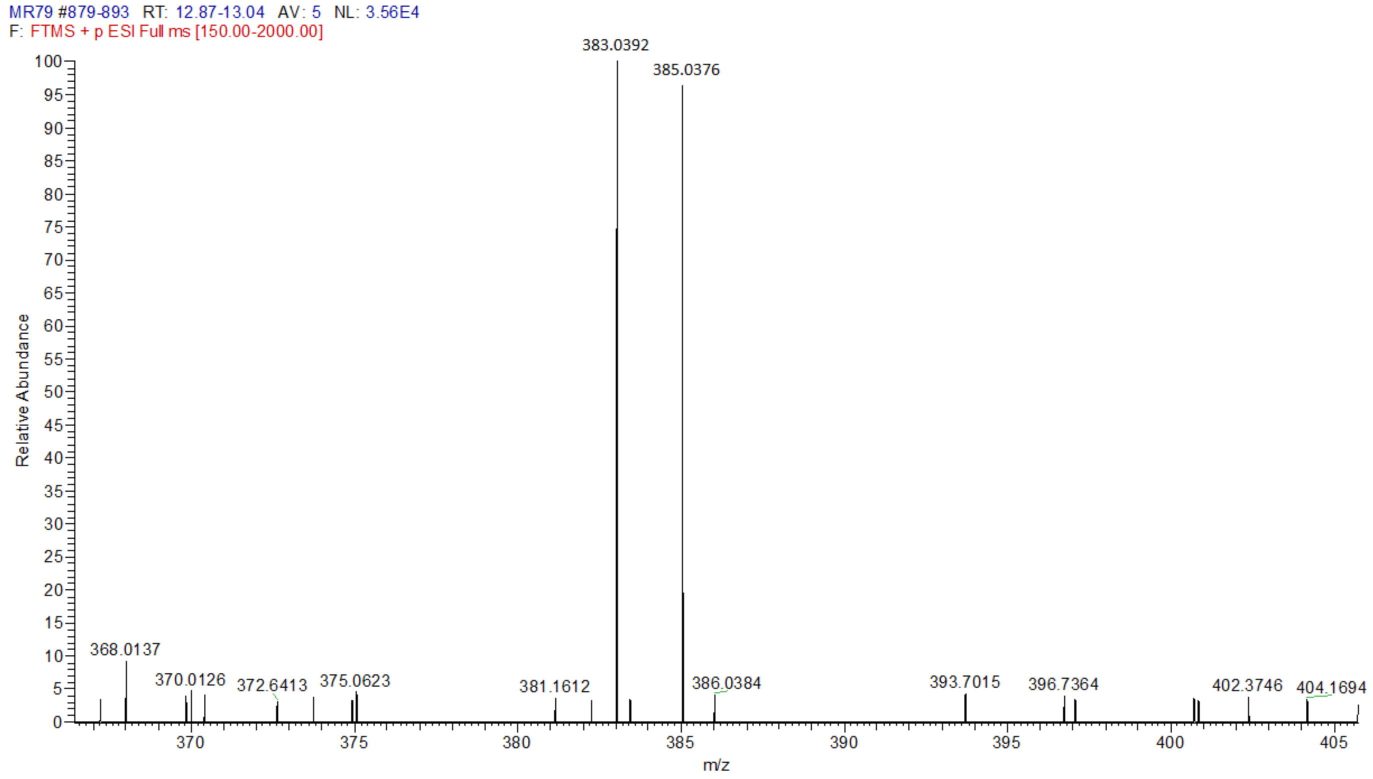

Fig.S1. HRESIMS spectrum of compound **1**

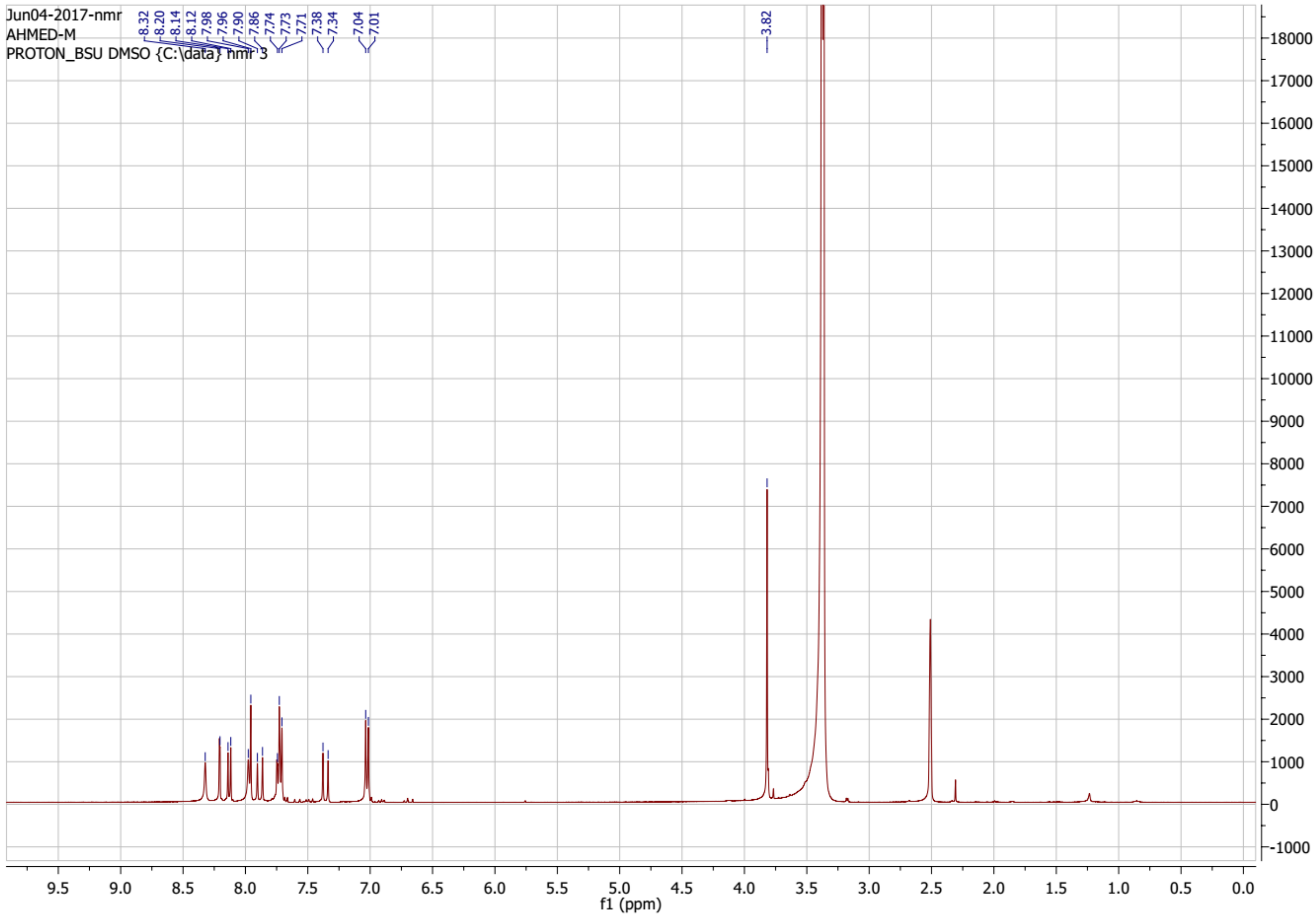

Fig.S2. ^{1}H NMR (400 MHz, DMSO-d_6) spectrum of **1**

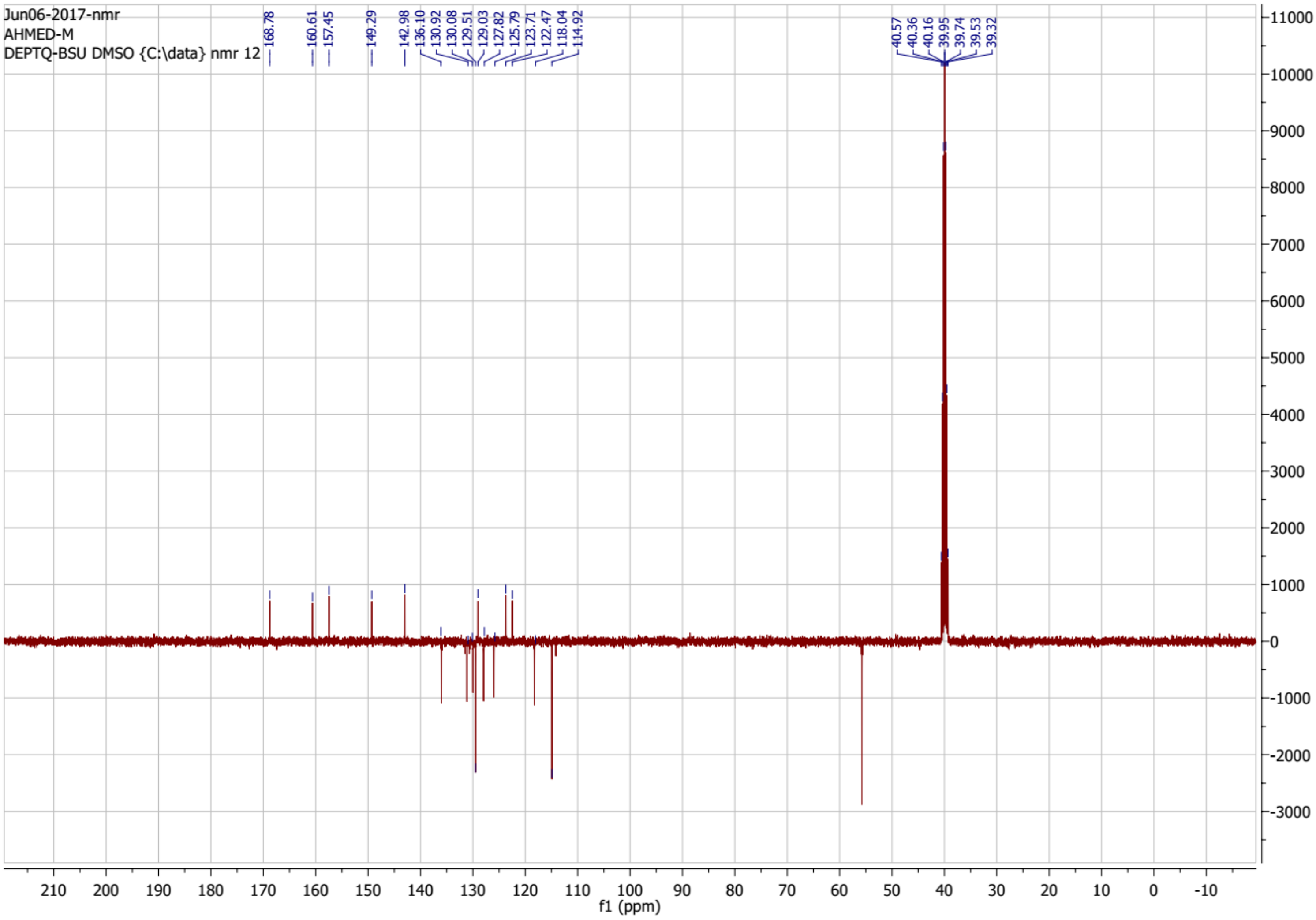

Fig.S3. DEPTQ (100 MHz, DMSO-d_6) spectrum of **1**

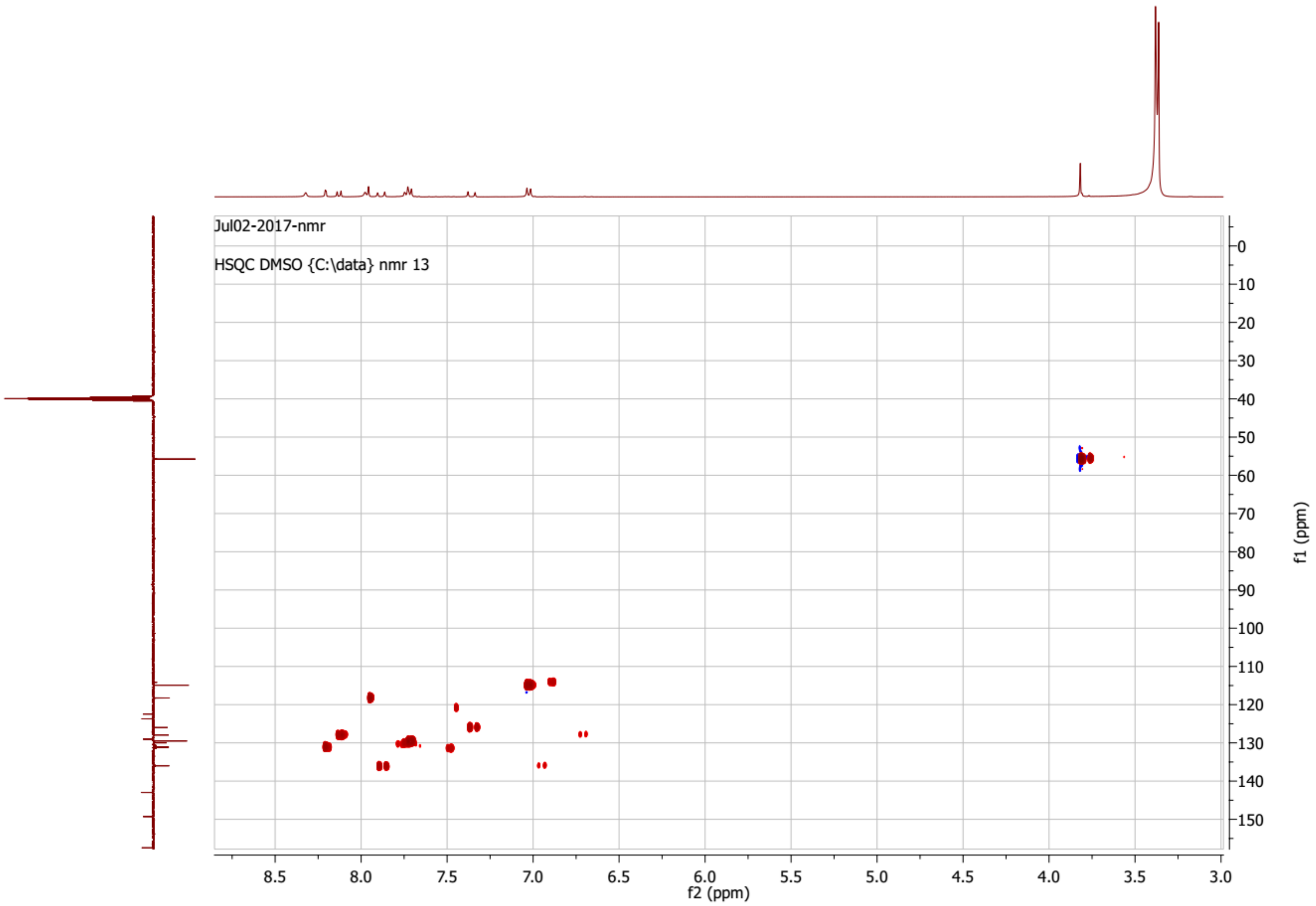

Fig.S4. HSQC (400 MHz, DMSO-d_6) spectrum of **1**

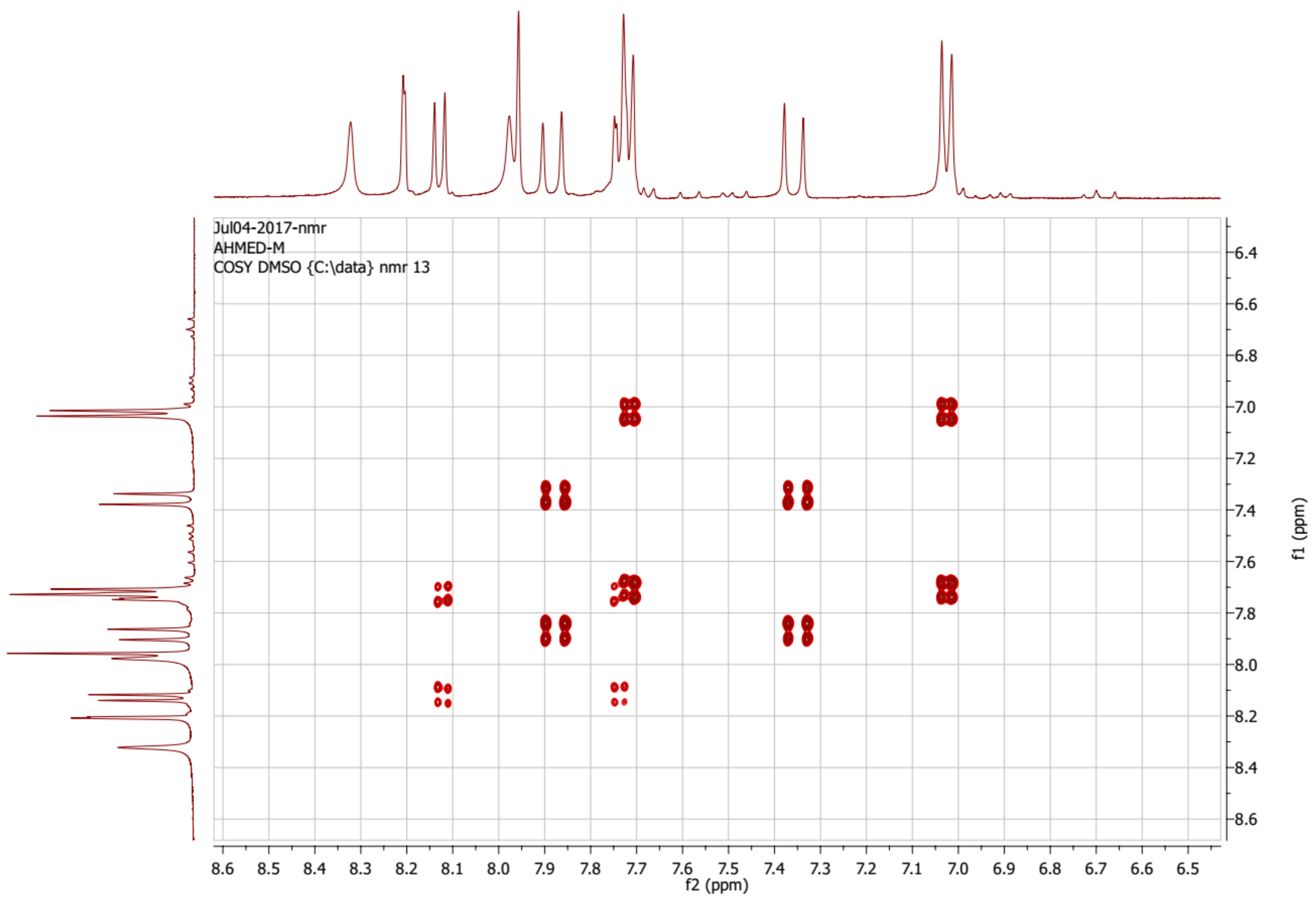

Fig.S5. ^{1}H-^{1}H COSY (400 MHz, DMSO-d_6) spectrum of **1**

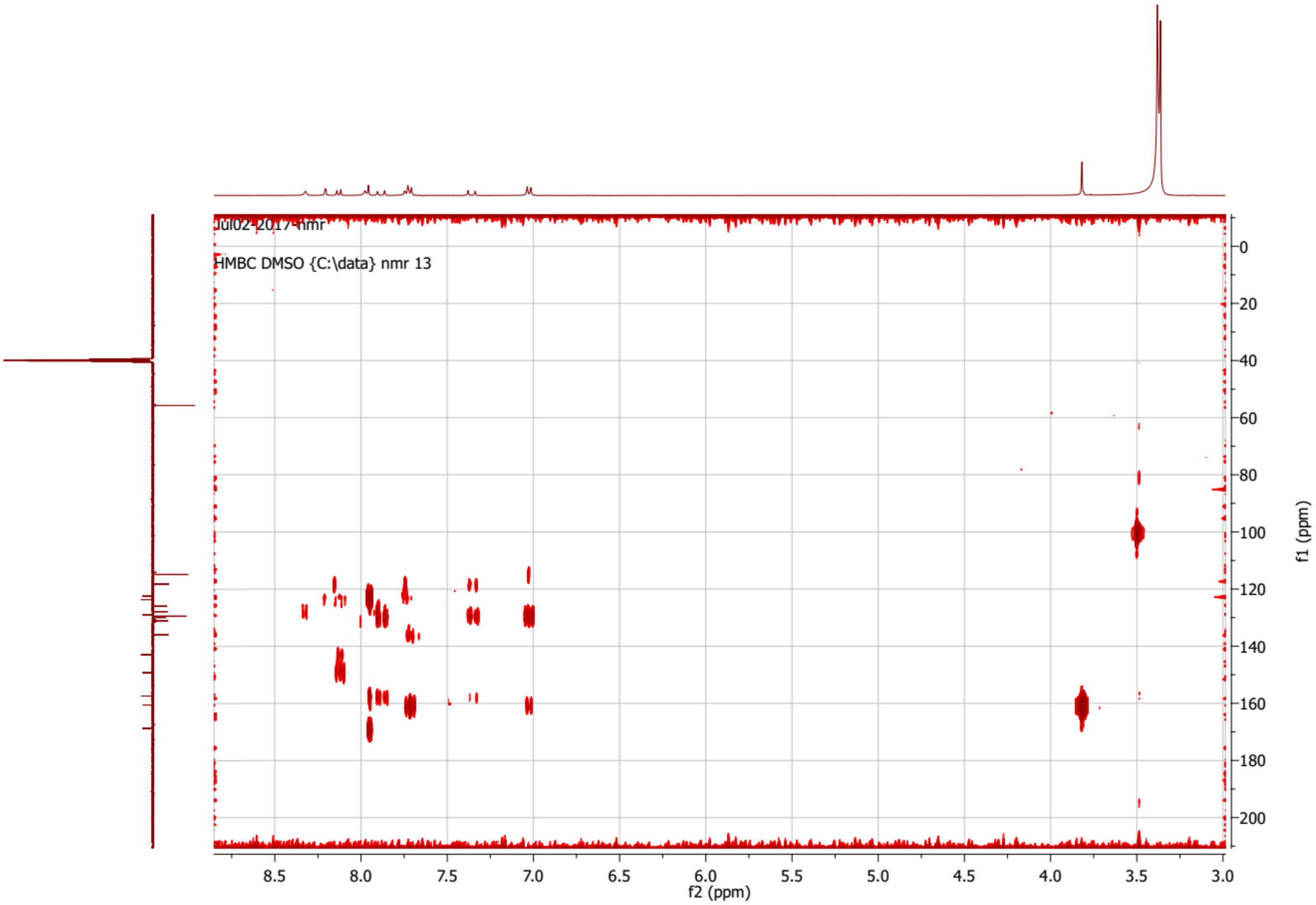

Fig.S6. HMBC (400 MHz, DMSO-d_6) spectrum of **1**

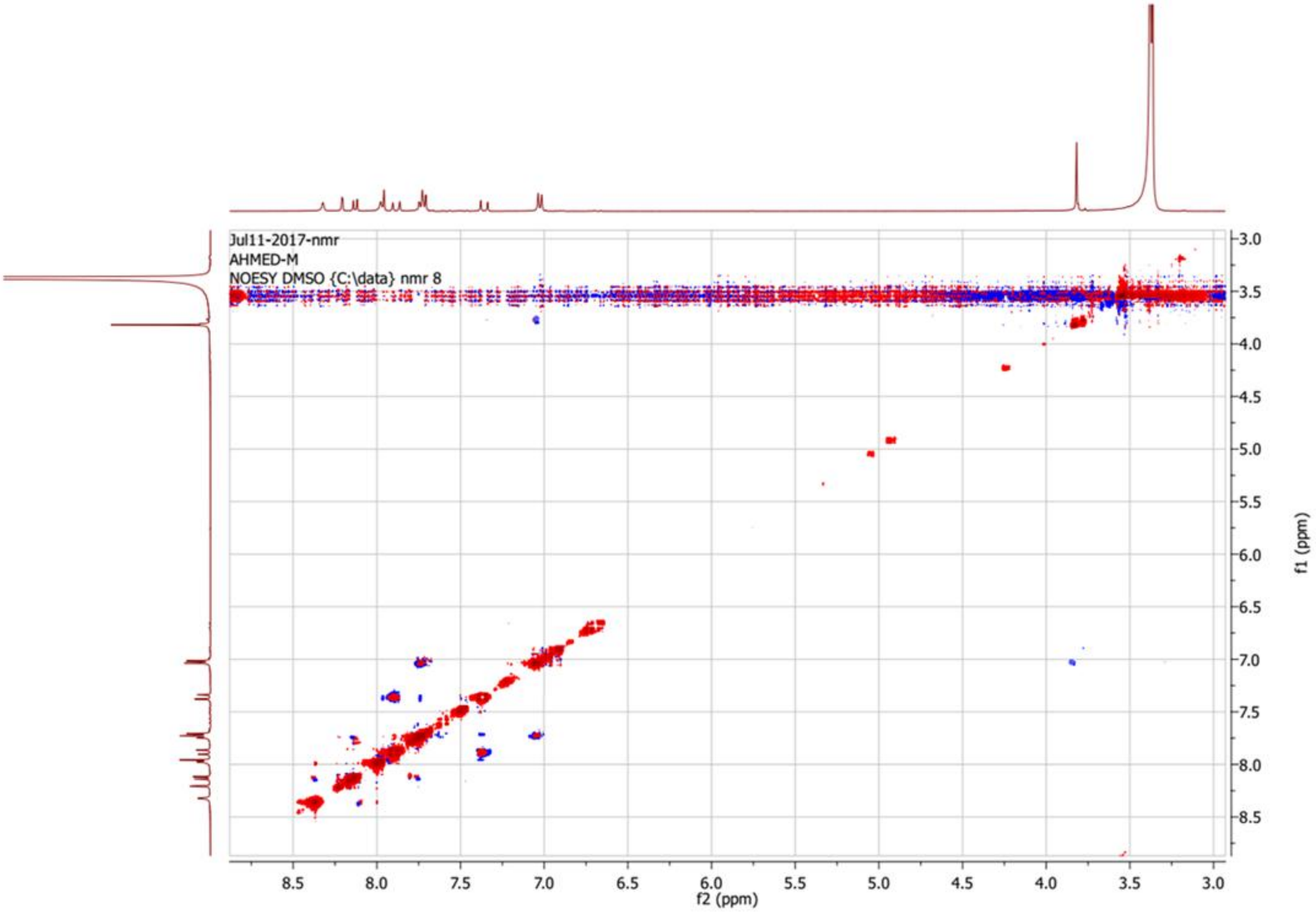

Fig.S7. NOESY (400 MHz, DMSO-d_6) spectrum of **1**

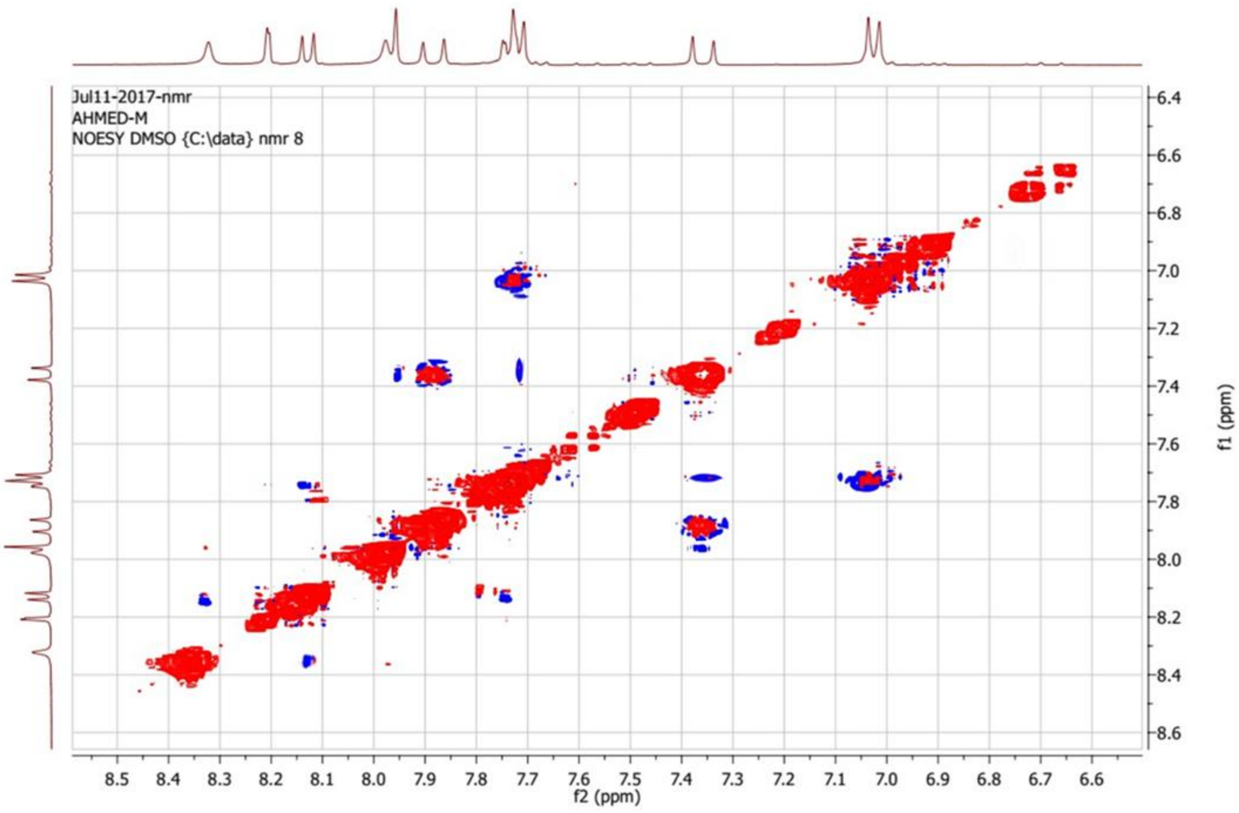

Fig.S8. NOESY expansion (400 MHz, DMSO-d_6) spectrum of **1**

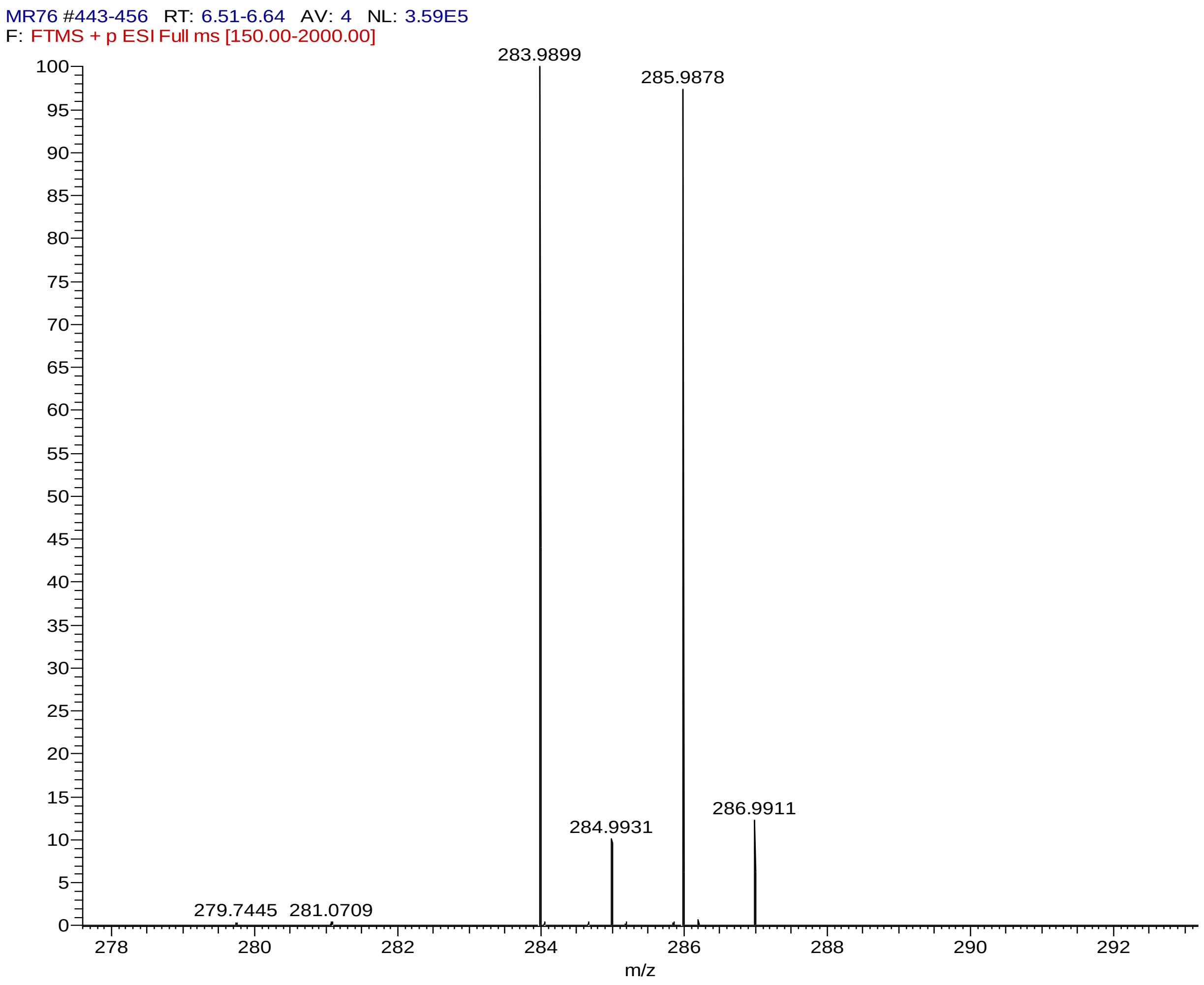

Fig.S9. HRESIMS spectrum of compound **2**

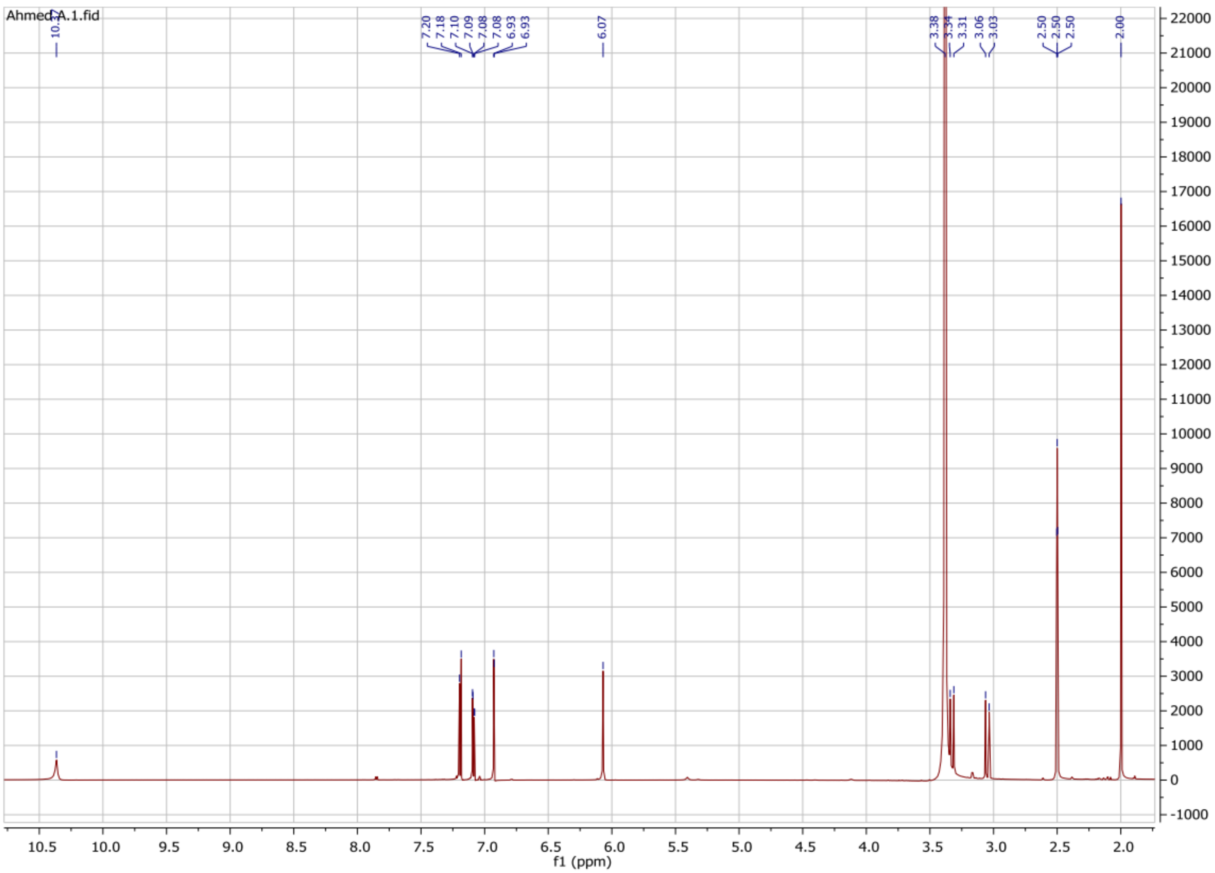

Fig.S10. ^{1}H NMR (400 MHz, DMSO-d_6) spectrum of **2**

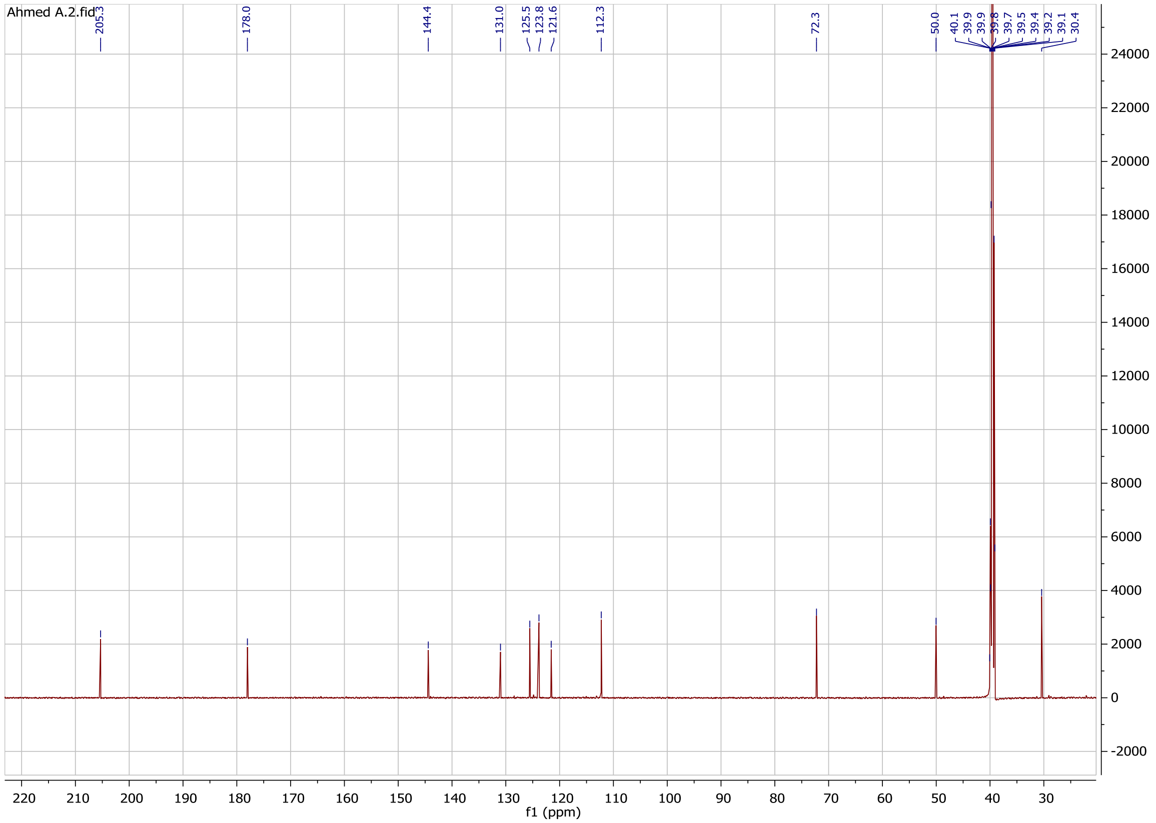

Fig.S11. ^{13}C NMR (400 MHz, DMSO-d_6) spectrum of **2**

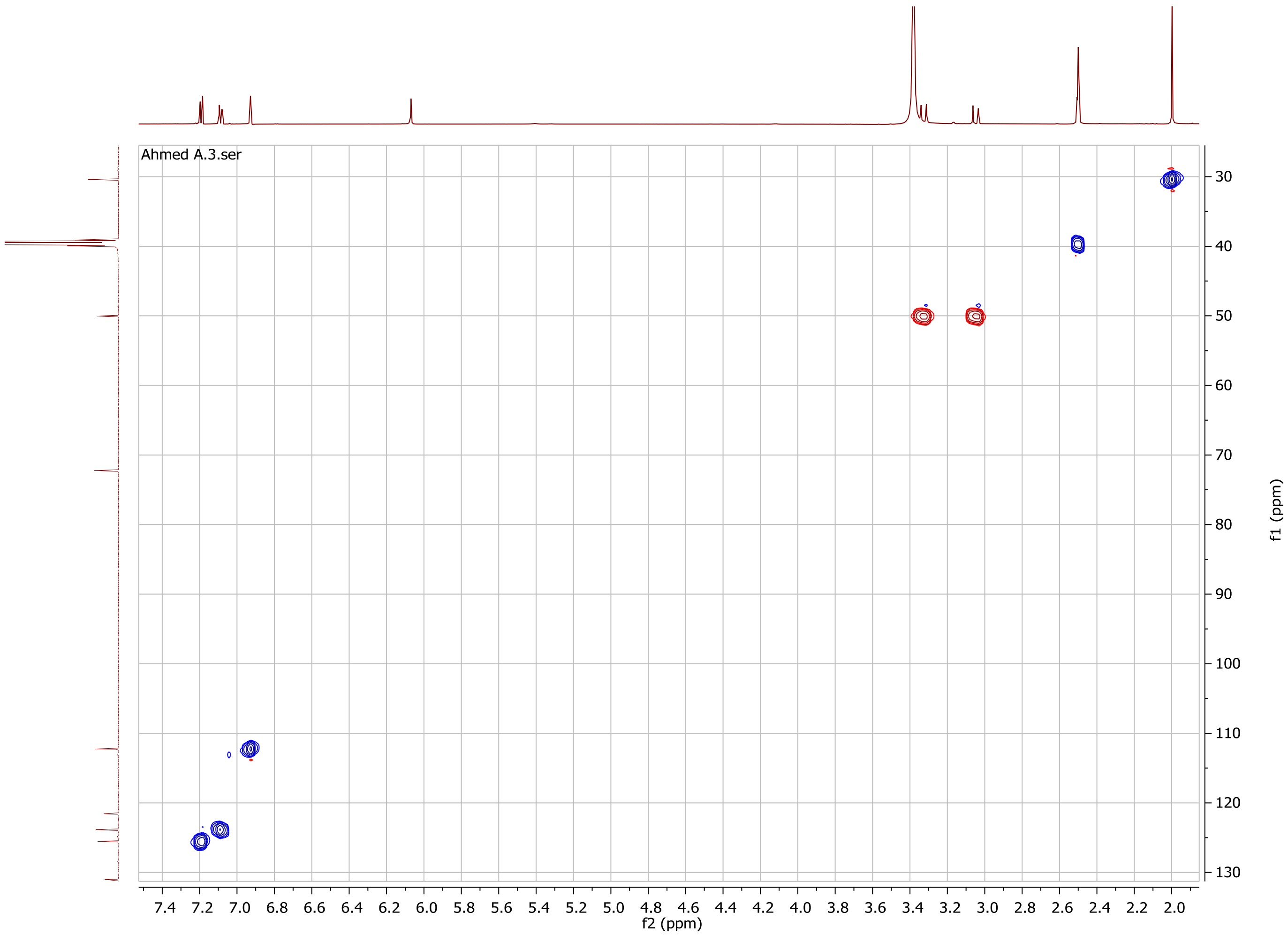

Fig.S12. HSQC (400 MHz, DMSO-d_6) spectrum of **2**

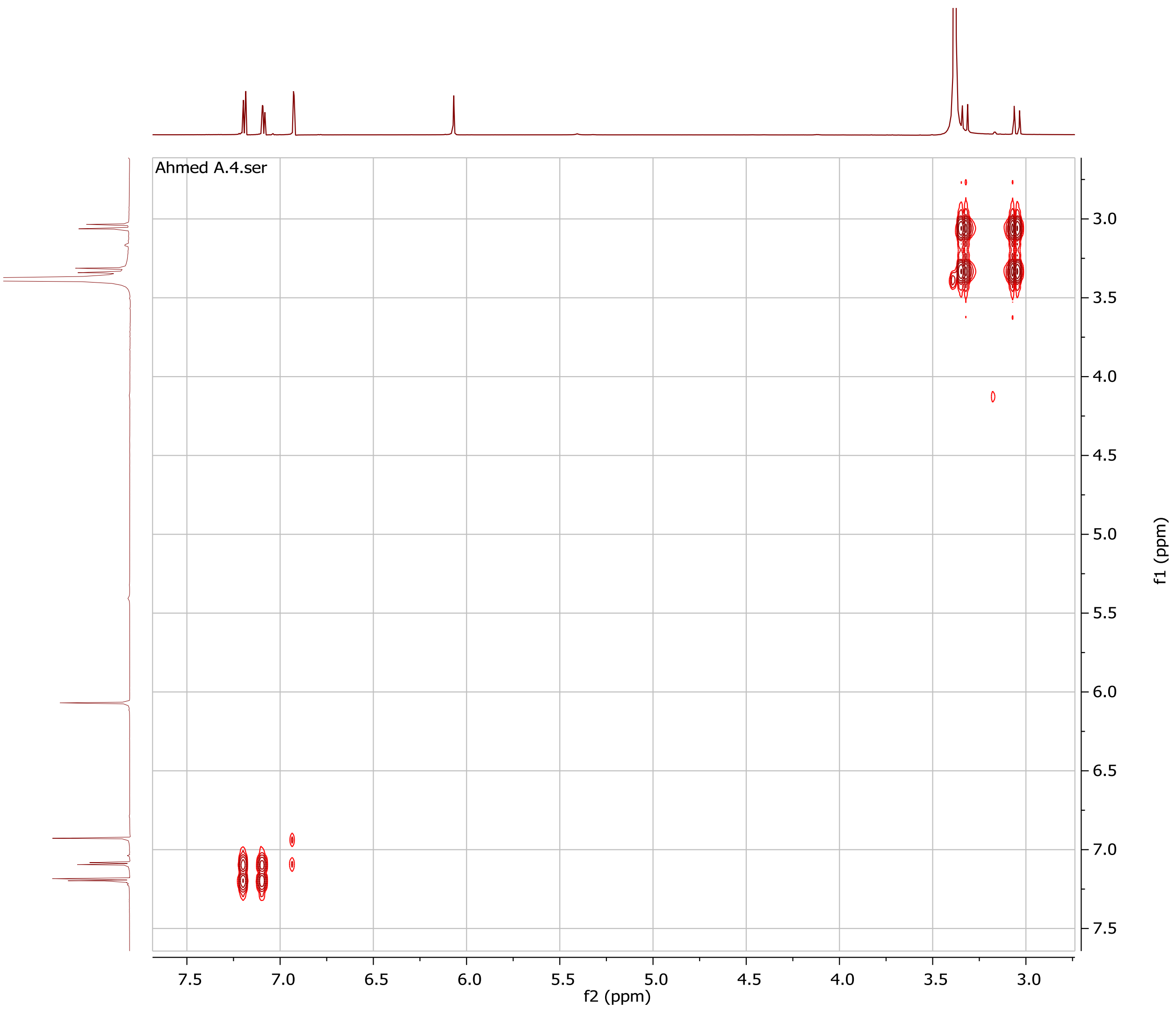

Fig.S13. ^{1}H-^{1}H COSY (400 MHz, DMSO-d_6) spectrum of **2**

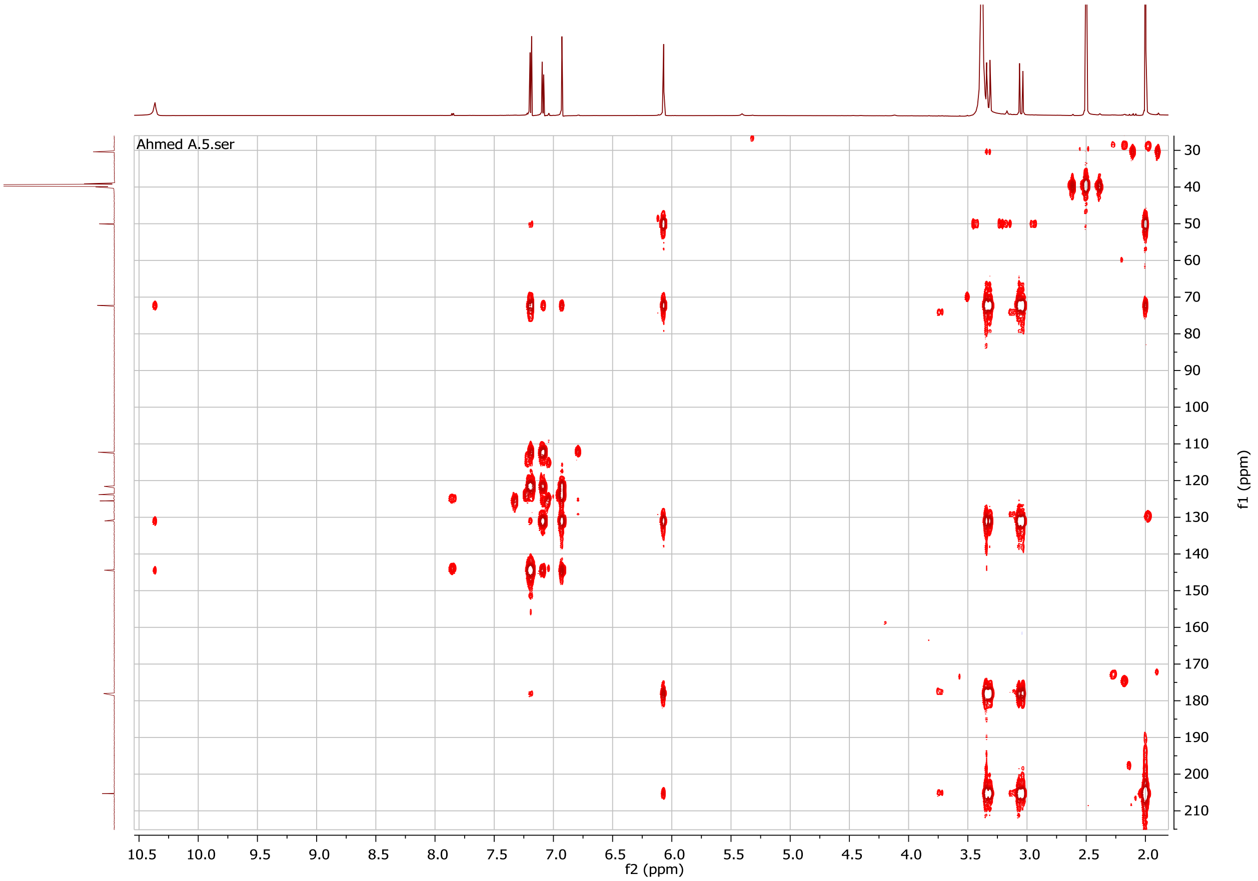

Fig.S14. HMBC (400 MHz, DMSO-d_6) spectrum of **2**

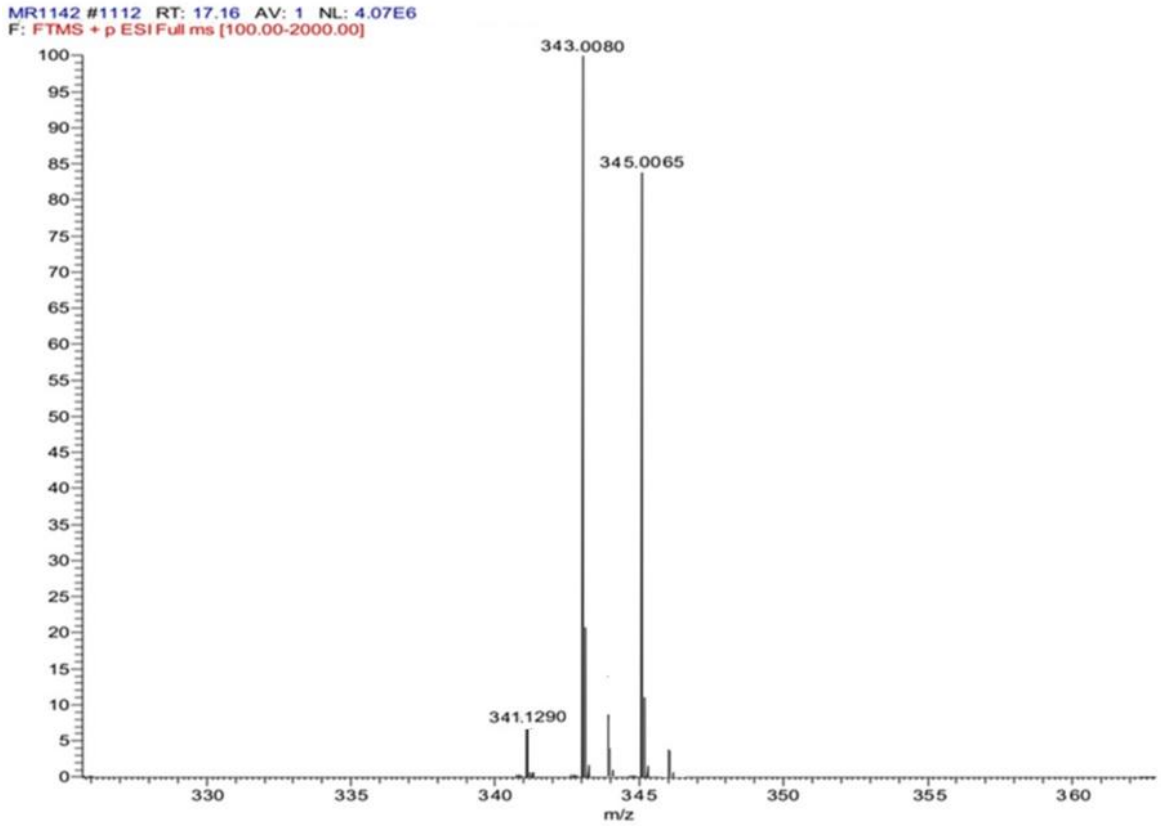

Fig.S15. HRESIMS spectrum of compound **3**

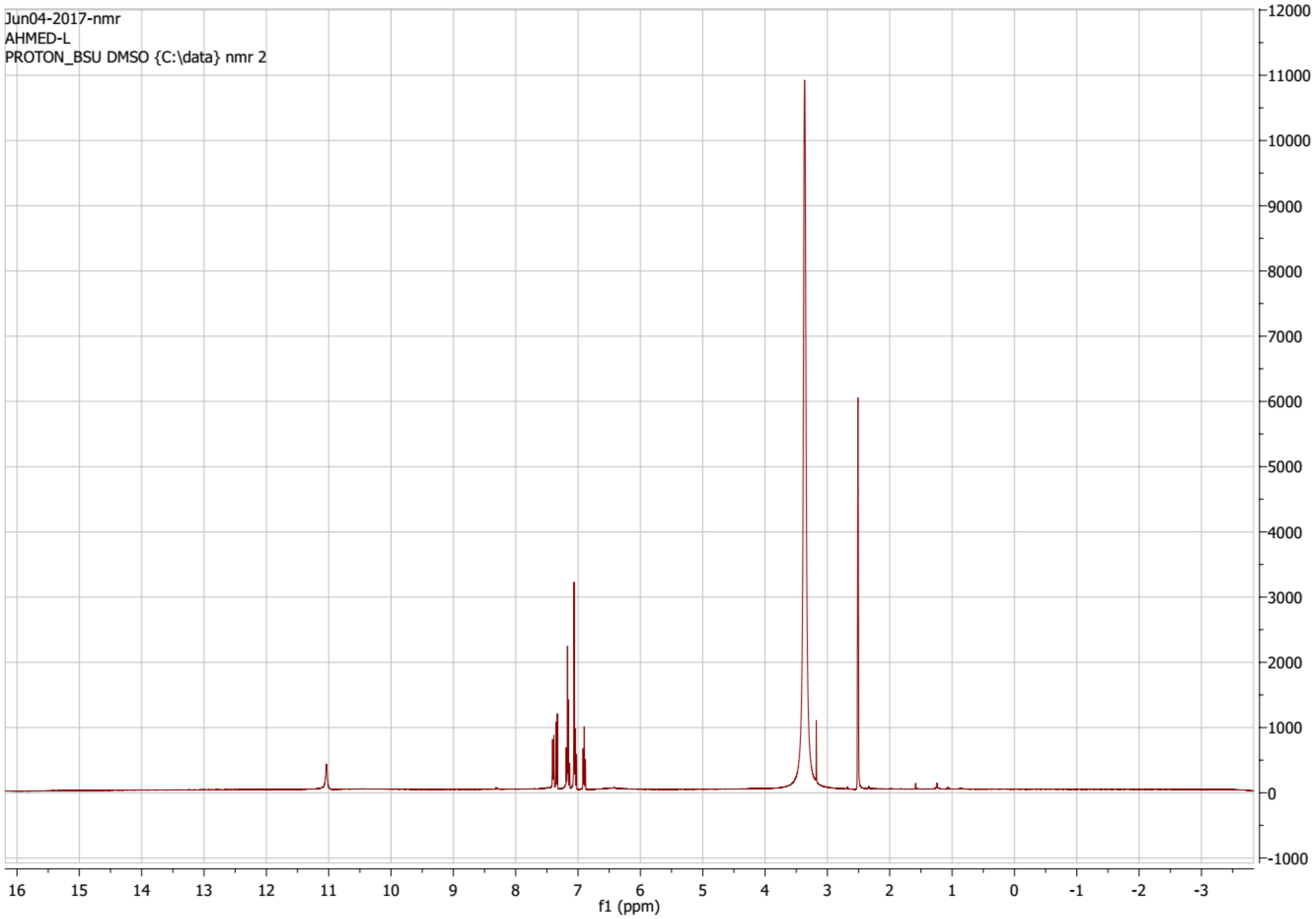

Fig.S16. ^{1}H NMR (400 MHz, DMSO-d_6) spectrum of **3**

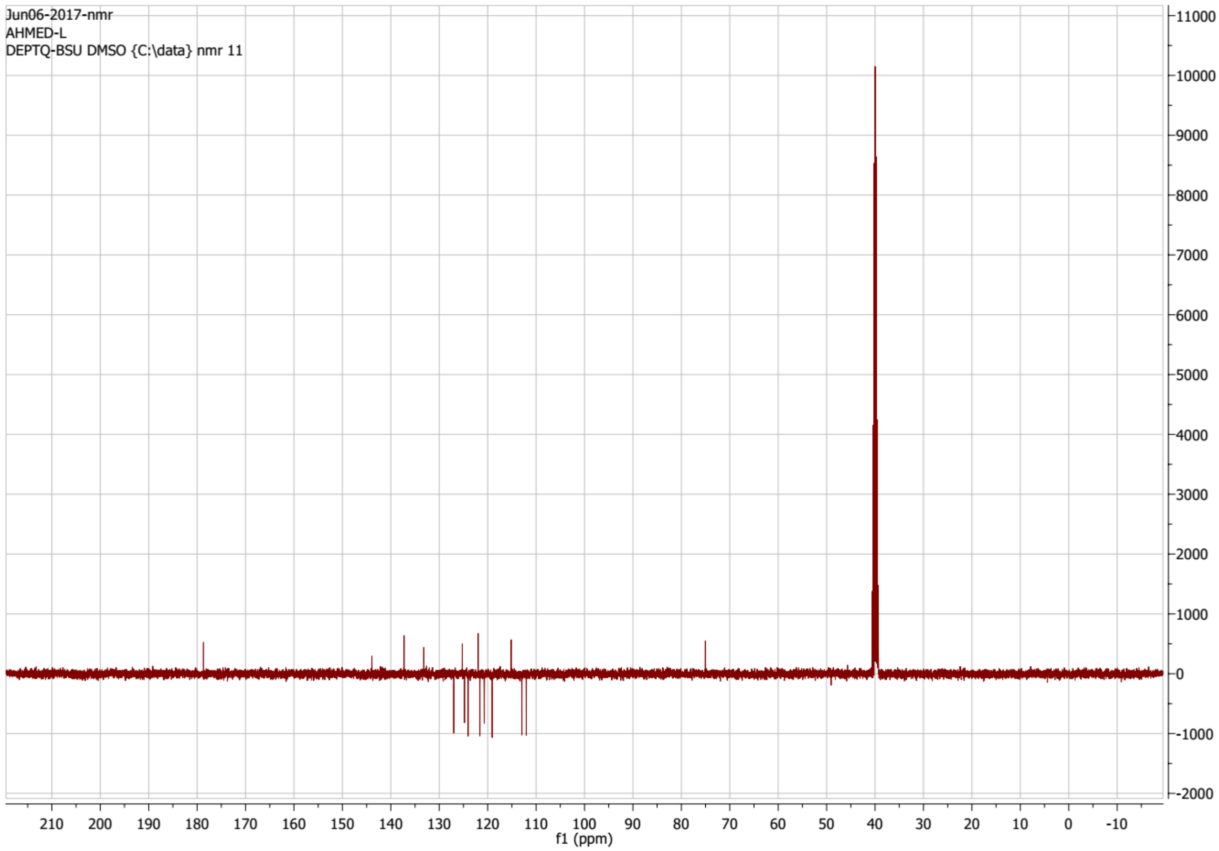

Fig.S17. DEPTQ NMR (400 MHz, DMSO-d_6) spectrum of **3**

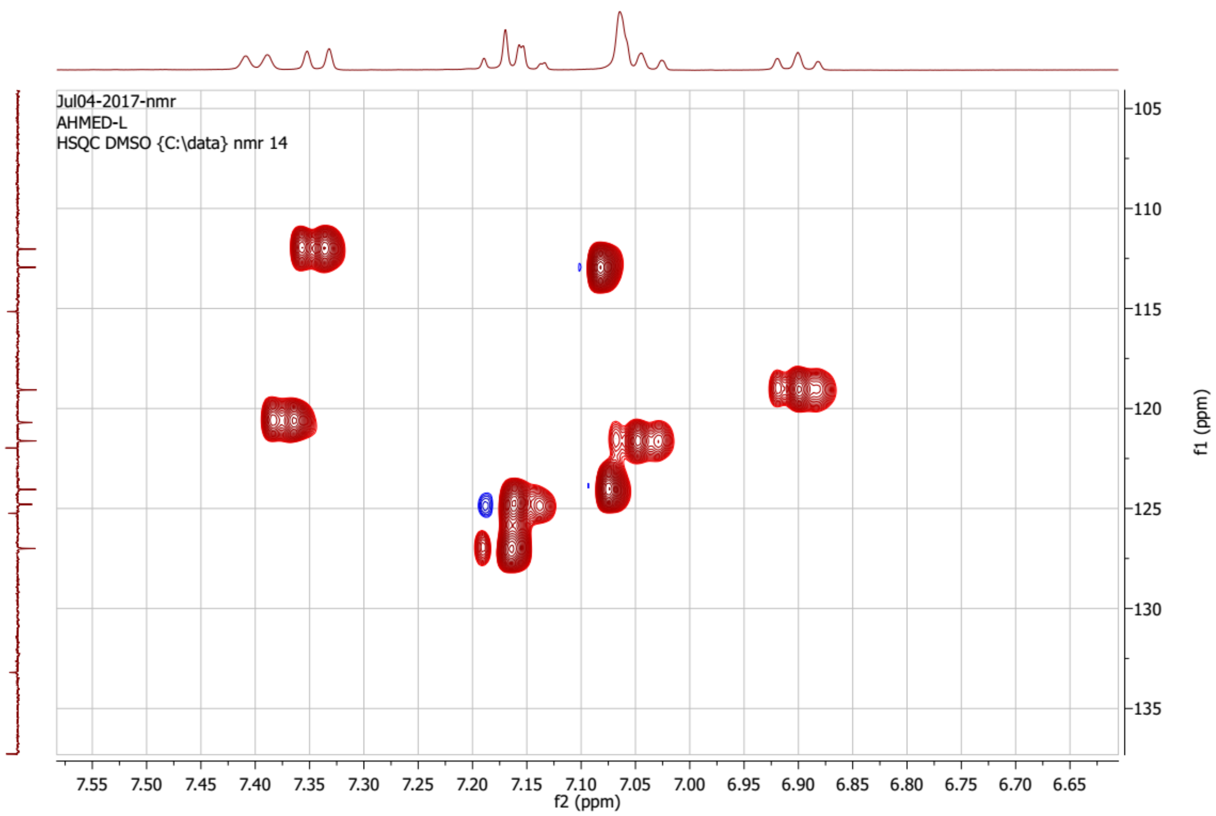

Fig.S18. HSQC (400 MHz, DMSO-d_6) spectrum of **3**

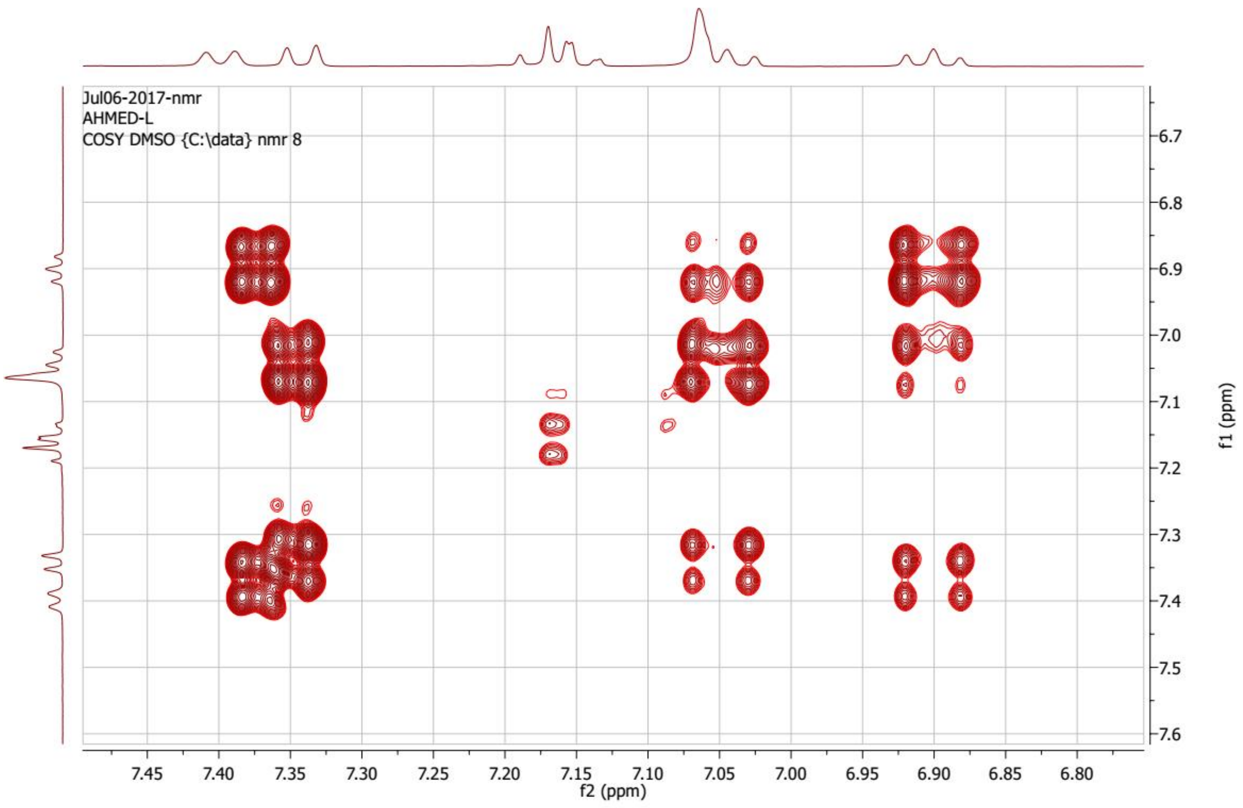

Fig.S19. ^{1}H-^{1}H COSY (400 MHz, DMSO-d_6) spectrum of **3**

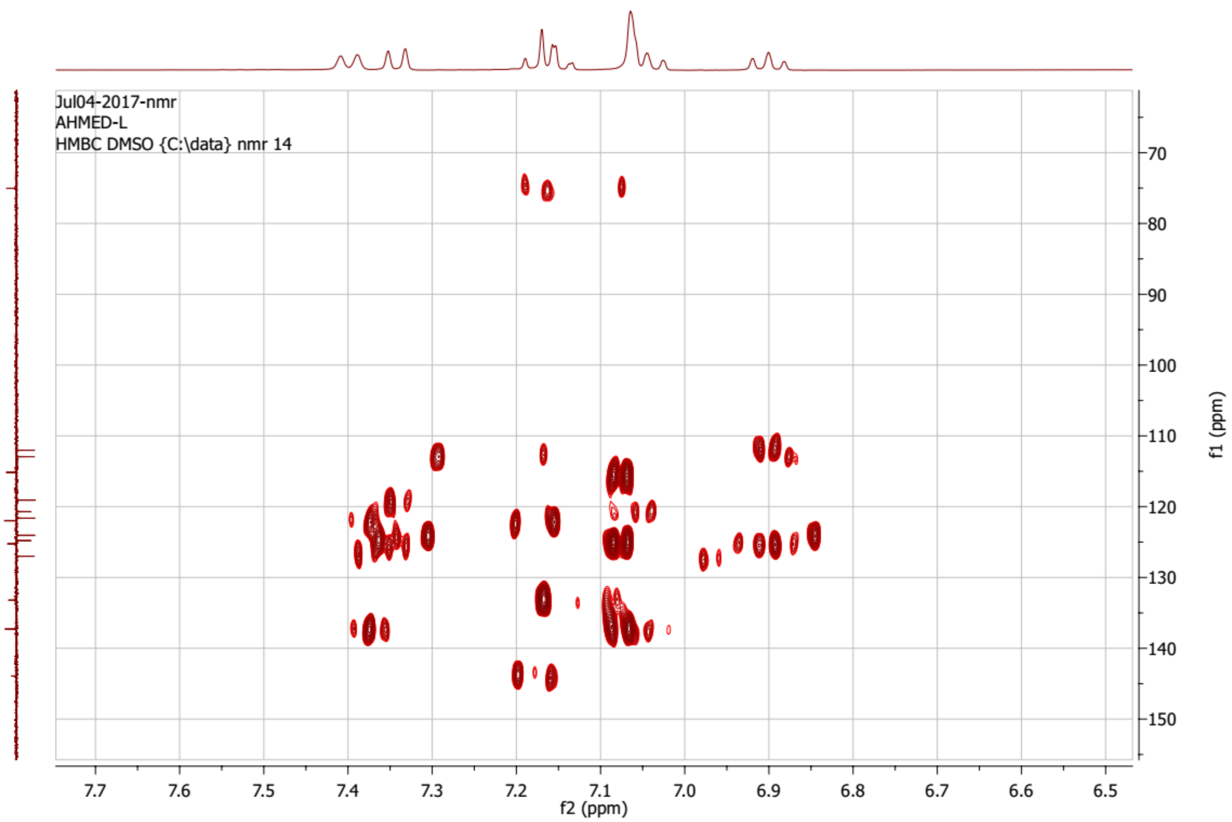

Fig.S20. HMBC (400 MHz, DMSO-d_6) spectrum of **3**

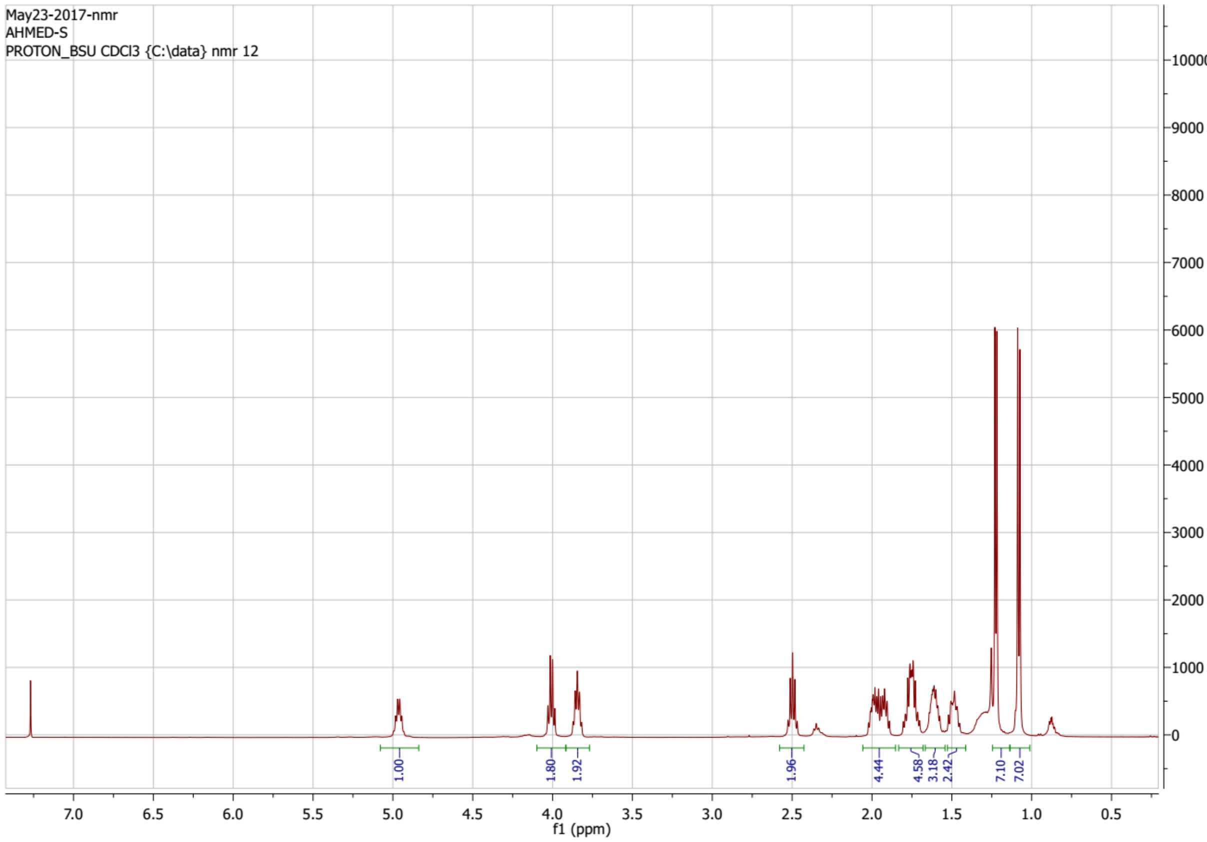

Fig.S21. ^{1}H NMR (400 MHz, $CDCl_3$) spectrum of **4**

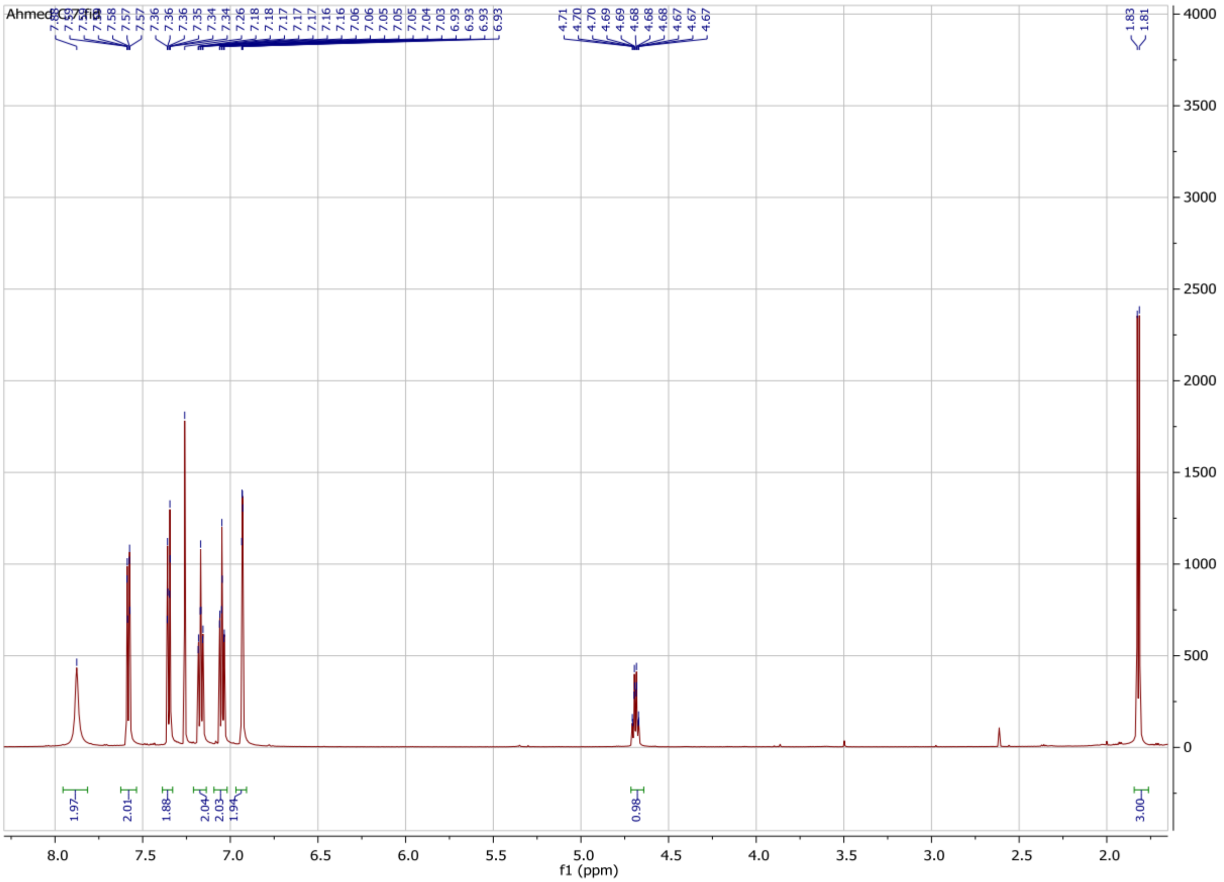

Fig.S22. ^{1}H NMR (400 MHz, $CDCl_3$) spectrum of **5**

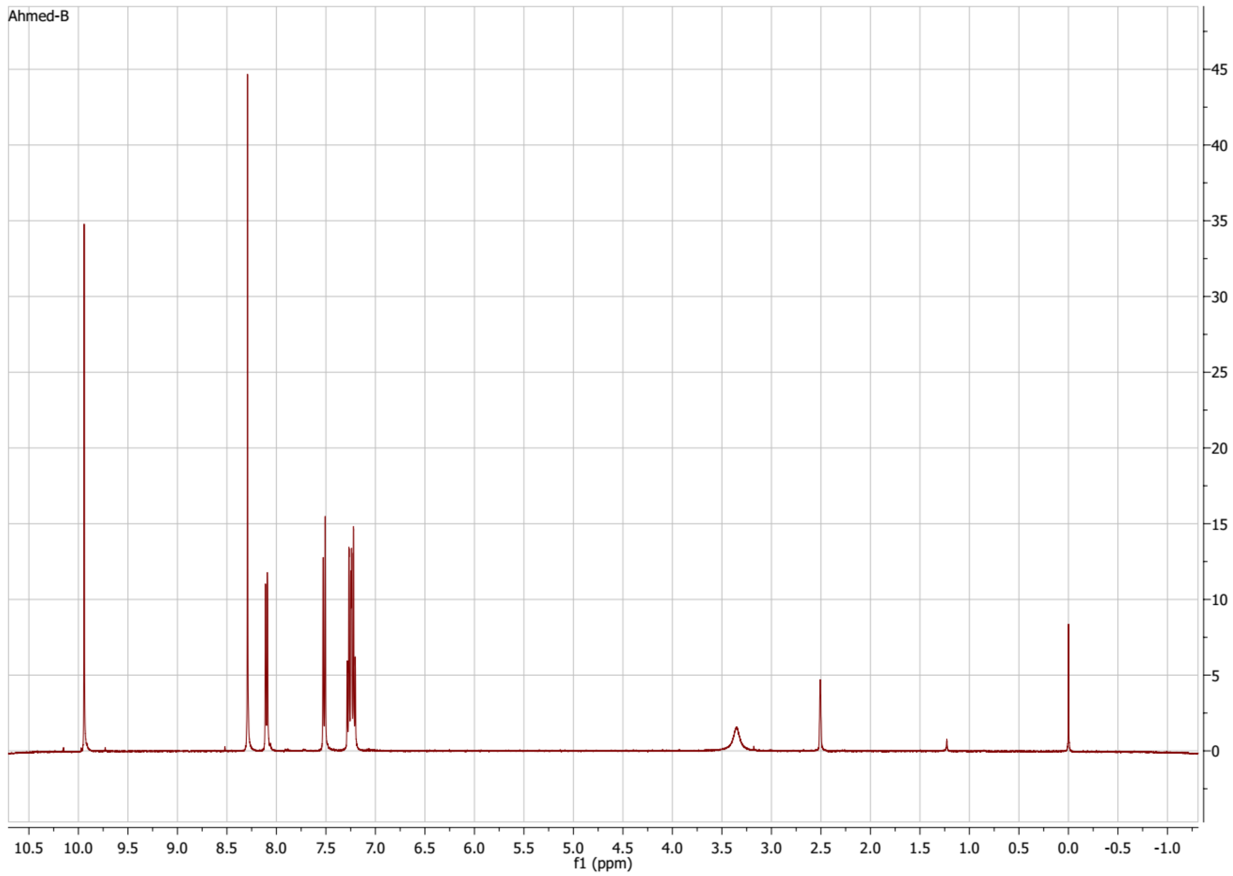

Fig.S23. ^{1}H NMR (400 MHz, DMSO-d_6) spectrum of **6**

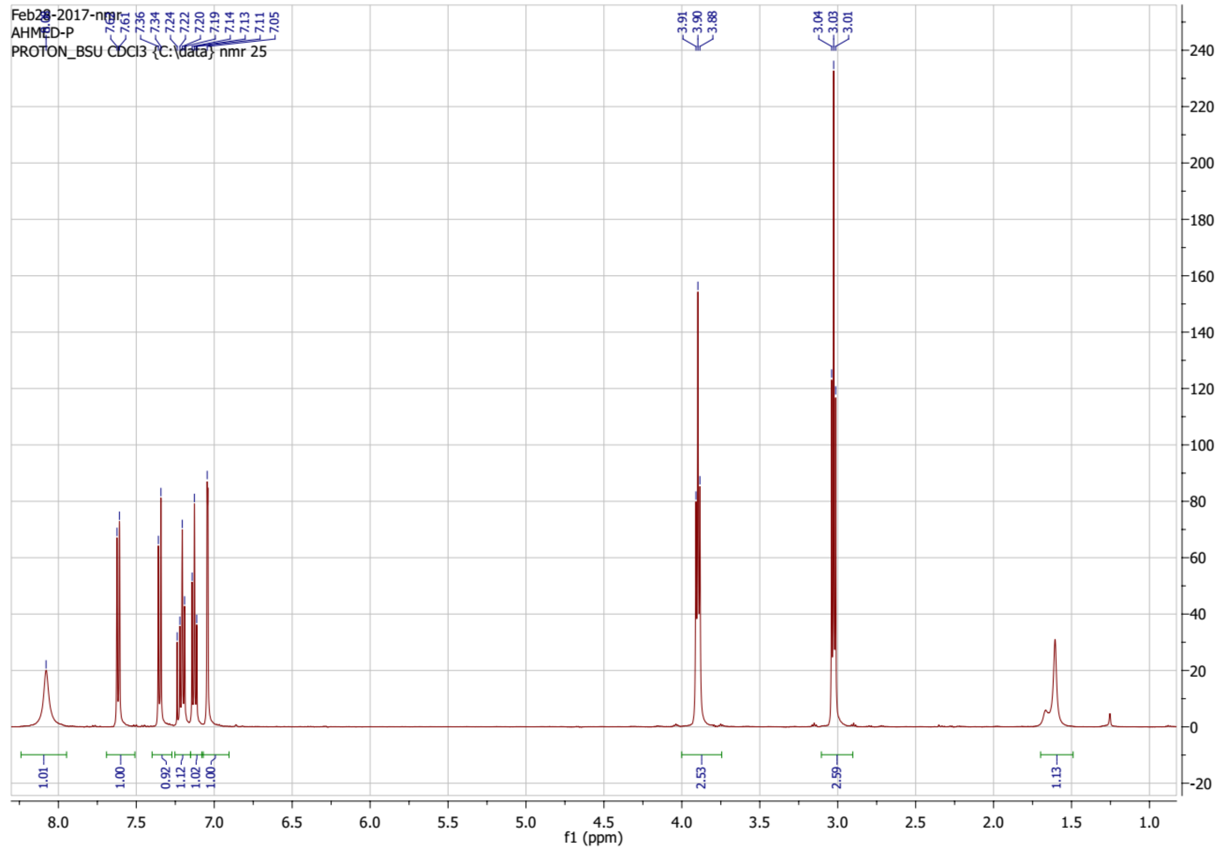

Fig.S24. ^{1}H NMR (400 MHz, $CDCl_3$) spectrum of **7**

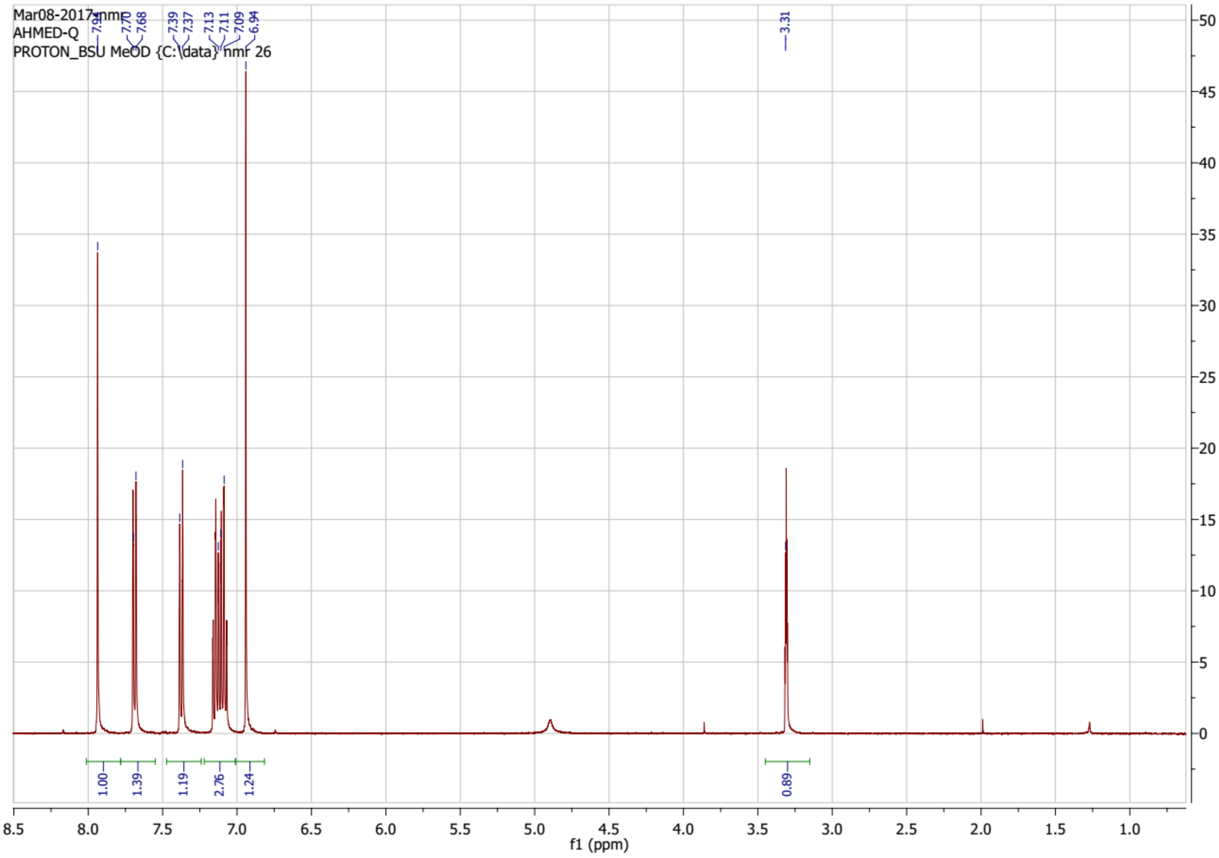

Fig.S25. ^{1}H NMR (400 MHz, CD_3OD) spectrum of **8**

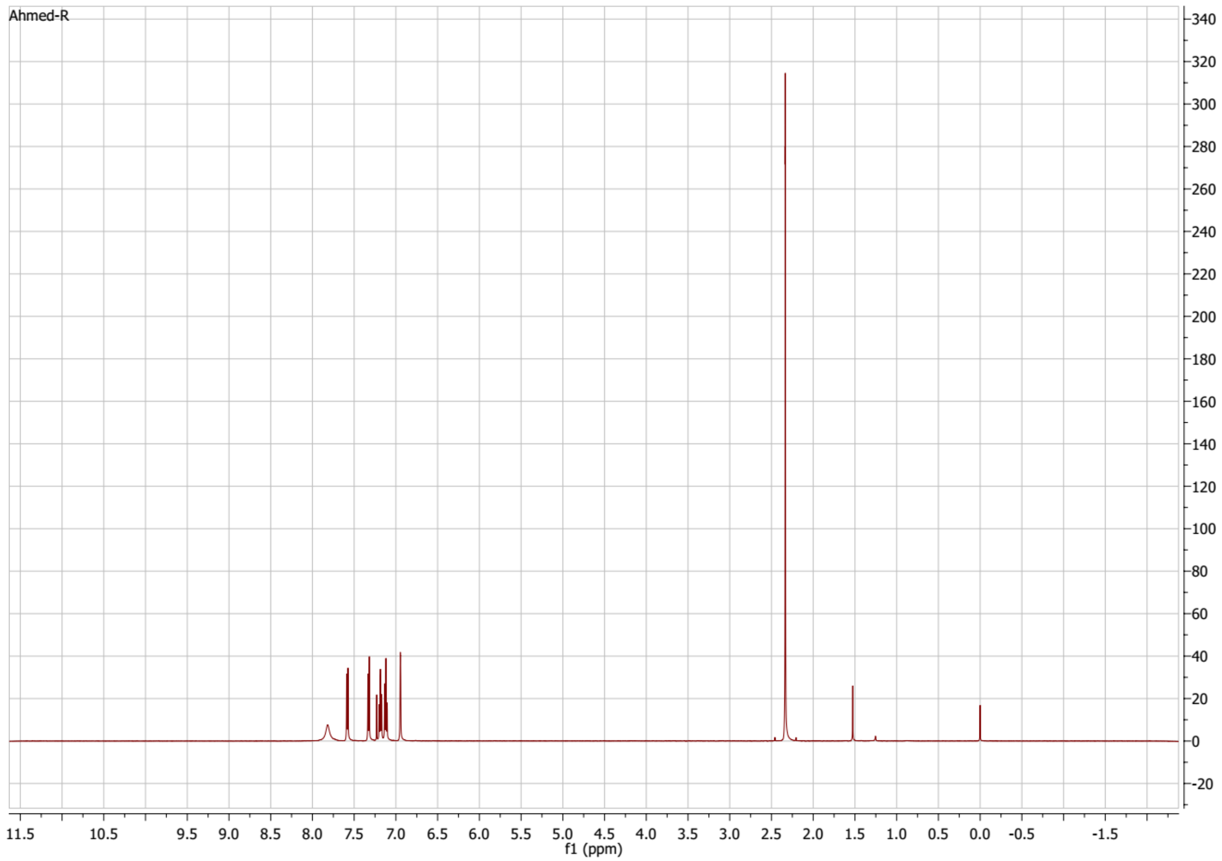

Fig.S26. ^{1}H NMR (400 MHz, $CDCl_3$) spectrum of **9**

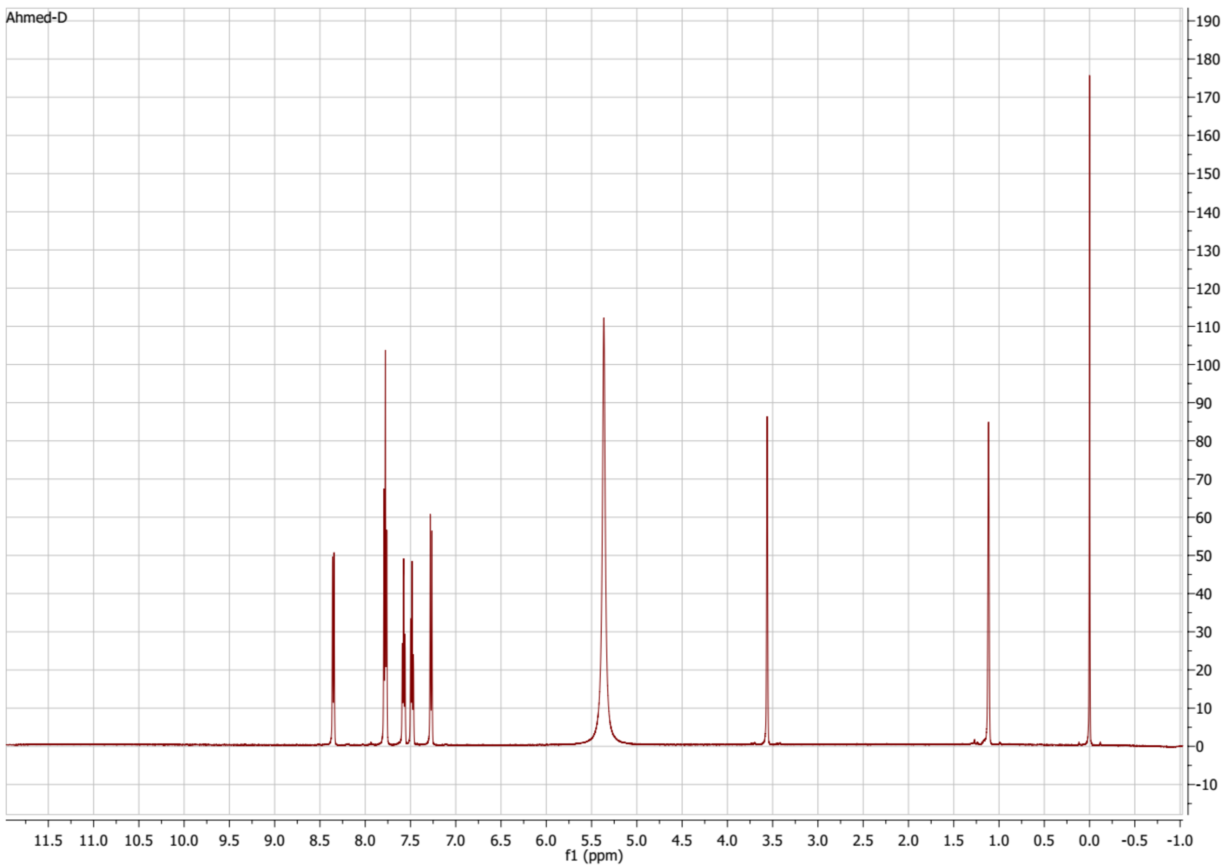

Fig.S27. ^{1}H NMR (400 MHz, CD_3OD) spectrum of **10**

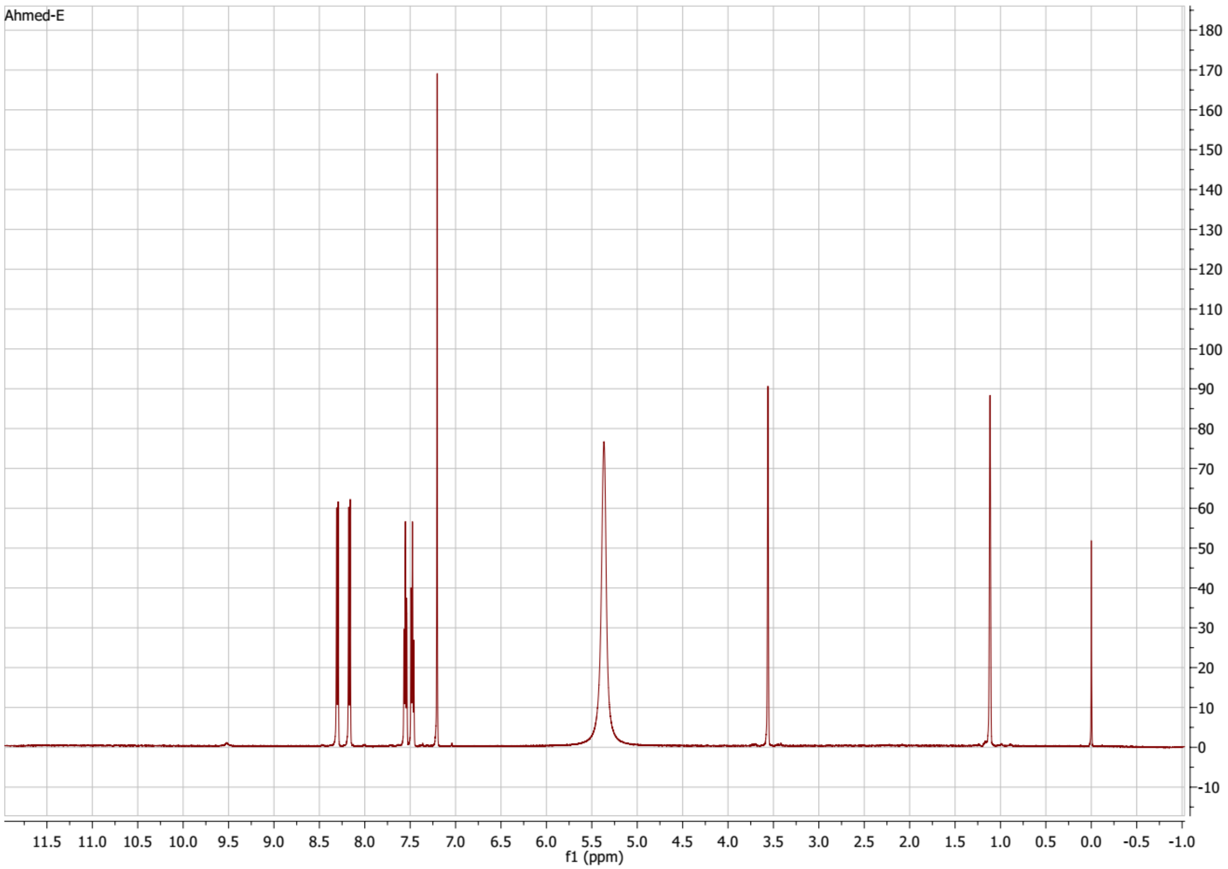

Fig.S28. ^{1}H NMR (400 MHz, CD_3OD) spectrum of **11**
